# Individual components of the SWI/SNF chromatin remodelling complex have distinct roles in memory neurons of the *Drosophila* mushroom body

**DOI:** 10.1242/dmm.037325

**Published:** 2019-03-25

**Authors:** Melissa C. Chubak, Kevin C. J. Nixon, Max H. Stone, Nicholas Raun, Shelby L. Rice, Mohammed Sarikahya, Spencer G. Jones, Taylor A. Lyons, Taryn E. Jakub, Roslyn L. M. Mainland, Maria J. Knip, Tara N. Edwards, Jamie M. Kramer

**Affiliations:** 1 Department of Biology, Faculty of Science, Western University, London, ON N6A 5B7, Canada; 2 Department of Physiology and Pharmacology, Schulich School of Medicine and Dentistry, Western University, London, ON N6A 5C1, Canada; 3 Division of Genetics and Development, Children's Health Research Institute, London, ON N6C 2V5, Canada

**Keywords:** SWI/SNF complex, *Drosophila melanogaster*, Mushroom body, Memory, Neuron remodelling, Intellectual disability

## Abstract

Technology has led to rapid progress in the identification of genes involved in neurodevelopmental disorders such as intellectual disability (ID), but our functional understanding of the causative genes is lagging. Here, we show that the SWI/SNF chromatin remodelling complex is one of the most over-represented cellular components disrupted in ID. We investigated the role of individual subunits of this large protein complex using targeted RNA interference in post-mitotic memory-forming neurons of the *Drosophila* mushroom body (MB). Knockdown flies were tested for defects in MB morphology, short-term memory and long-term memory. Using this approach, we identified distinct roles for individual subunits of the *Drosophila* SWI/SNF complex. Bap60, Snr1 and E(y)3 are required for pruning of the MBγ neurons during pupal morphogenesis, while Brm and Osa are required for survival of MBγ axons during ageing. We used the courtship conditioning assay to test the effect of MB-specific SWI/SNF knockdown on short- and long-term memory. Several subunits, including Brm, Bap60, Snr1 and E(y)3, were required in the MB for both short- and long-term memory. In contrast, Osa knockdown only reduced long-term memory. Our results suggest that individual components of the SWI/SNF complex have different roles in the regulation of structural plasticity, survival and functionality of post-mitotic MB neurons. This study highlights the many possible processes that might be disrupted in SWI/SNF-related ID disorders. Our broad phenotypic characterization provides a starting point for understanding SWI/SNF-mediated gene regulatory mechanisms that are important for development and function of post-mitotic neurons.

## Abstract (precis)

**Summary:** The SWI/SNF chromatin remodelling complex is the most over-represented protein complex in the intellectual disability. Different components of this complex have distinct roles in development and function of memory-forming neurons in the *Drosophila* mushroom body.

## INTRODUCTION

Intellectual disability (ID) is a neurodevelopmental disorder characterized by early-onset limitations in cognitive function and adaptive behaviour that affects 1-3% of the global population. Technological advances in DNA sequencing have led to rapid progress in understanding the genetic aetiology of ID, and, as a result, there are currently ∼1000 known primary ID genes (sysid.cmbi.umcn.nl) (Kochinke et al., 2016). For most of these genes, we have no knowledge of their role in the nervous system. Therefore, functional analysis of known ID genes is the next frontier in advancing our understanding of neurodevelopmental disorders.

About two-thirds of known ID genes follow a recessive or X-linked inheritance pattern. Historically, such inheritance patterns have made it possible to identify disease genes using family pedigrees combined with genomic methodologies, such as homozygosity mapping. However, recent studies suggest that recessive and X-linked inheritance patterns are not representative of the majority of ID cases (de Ligt et al., 2012; Gilissen et al., 2014; Rauch et al., 2012; The Deciphering Developmental Disorders Study, 2014; Veltman and Brunner, 2012; Vissers et al., 2010). In fact, most people with ID have a dominant genetic variant that is acquired *de novo*. Dominant *de novo* copy-number variations and single-nucleotide variants are estimated to account for 60% of severe ID cases, compared to only 2% of severe ID cases that are caused by rare inherited forms (Gilissen et al., 2014). Several large-scale studies on cohorts of patients with variable clinical presentation paint a similar picture, with a prominent role for dominant ID genes (DIGs) (de Ligt et al., 2012; Hamdan et al., 2014; Rauch et al., 2012; The Deciphering Developmental Disorders Study, 2014).

Recently, all known ID genes have been documented in a hand-curated publicly accessible database called sysID (sysid.cmbi.umcn.nl) (Kochinke et al., 2016). There are 339 DIGs documented in this database (updated December 2017). Here, we show that DIGs are highly cohesive, suggesting that they may be involved in common pathways or biological processes. We find that DIGs are enriched for genes encoding proteins associated with chromatin regulation and identify the SWI/SNF ATP-dependent chromatin remodelling complex as the most enriched DIG-associated cellular component. Currently, mutations in 12 of the 29 genes that encode subunits of the human SWI/SNF complex have been found in patients with ID (Bramswig et al., 2017; Dias et al., 2016; Kleefstra et al., 2012; Machol et al., 2019; Marom et al., 2017; Nixon et al., 2018; Rivière et al., 2012; Tsurusaki et al., 2012; Van Houdt et al., 2012).

The SWI/SNF complex was identified in yeast and is highly conserved (Sudarsanam and Winston, 2000). Each conformation of the SWI/SNF complex contains 10-15 protein subunits, including a single ATPase that utilizes energy from ATP to alter nucleosome positioning, making chromatin either more or less accessible for interactions with transcription factors. The SWI/SNF complex can increase chromatin accessibility at cell-type-specific enhancers in human cancer cell lines and mouse embryonic fibroblasts (Alver et al., 2017; Vierbuchen et al., 2017; Wang et al., 2017), and is essential for maintaining global epigenetic programs required for cell-type specification, differentiation and neuronal stem cell proliferation during mouse brain development (Eroglu et al., 2014; Koe et al., 2014; Lessard et al., 2007; Narayanan et al., 2015; Sokpor et al., 2017; Tuoc et al., 2013). Double knockout of the paralogous SWI/SNF components BAF155 (also known as Smarcc1) and BAF170 (also known as Smarcc2) in mouse leads to proteasomal degradation of the entire complex, providing a unique system to investigate the consequences of complete loss of SWI/SNF function in neurons (Narayanan et al., 2015). In this scenario, it was shown that SWI/SNF is critical in neural progenitors for the specification of different brain structures (Bachmann et al., 2016; Narayanan et al., 2015).

These studies highlight the essential nature of SWI/SNF-mediated gene regulation in neuron differentiation; however, the mechanisms that are disrupted in ID are still not known. ID-causing SWI/SNF mutations are heterozygous and have been proposed to cause haploinsufficiency, dominant-negative or gain-of-function effects, depending on the nature of the mutation and the specific SWI/SNF subunit involved (Kosho et al., 2014; Santen et al., 2013, 2014; Van Houdt et al., 2012). It is likely that partial SWI/SNF function is maintained in individuals with these mutations. Therefore, despite the clear importance of SWI/SNF subunits in cell differentiation and tissue specification, human SWI/SNF-related disorders may result from more subtle defects in gene regulation in post-mitotic neurons. In mouse, the Baf53b (also known as Actl6b) SWI/SNF subunit is only incorporated into the complex in differentiated post-mitotic neurons. Deletion of this neuron-specific subunit does not affect complex assembly or neuron survival, but is required for activity-dependent dendritic outgrowth and long-term memory (Staahl et al., 2013; Vogel-Ciernia et al., 2013; Wu et al., 2007). Like Baf53b, other SWI/SNF components are also known to mediate specific gene regulatory mechanisms without interfering with the overall integrity of the complex (Tuoc et al., 2017; Weider et al., 2012). However, the role of most SWI/SNF complex components in post-mitotic neurons remains unexplored.

Here, we have systematically characterized the role of individual SWI/SNF subunits through targeted RNA interference (RNAi) in post-mitotic neurons of the *Drosophila* mushroom body (MB), a critical brain structure for learning and memory (de Belle and Heisenberg, 1994; McBride et al., 1999). The SWI/SNF complex is highly conserved in *Drosophila* and has recently been shown to assemble in distinct modules: a core module (Bap60, Snr1, Mor, Bap111), an ATPase module (Brm, Bap55, Actin), and one of two distinct ARID modules (Mashtalir et al., 2018). The two ARID modules distinguish the two established *Drosophila* SWI/SNF configurations, known as the Brahma-associated protein (BAP) and Polybromo BAP (PBAP) complexes (Mohrmann and Verrijzer, 2005). BAP and PBAP contain an overlapping set of subunits from the core and ATPase modules, as well as the complex-specific subunits: Osa and D4 for BAP, and Polybromo, Bap170 and E(y)3 for PBAP (Chalkley et al., 2008; Mashtalir et al., 2018; Mohrmann et al., 2004). We have identified unique requirements for individual SWI/SNF components in different aspects of MB neurobiology at different stages of fly development.

## RESULTS

### The SWI/SNF complex is the most enriched cellular component among DIGs

**Fig. 1.**
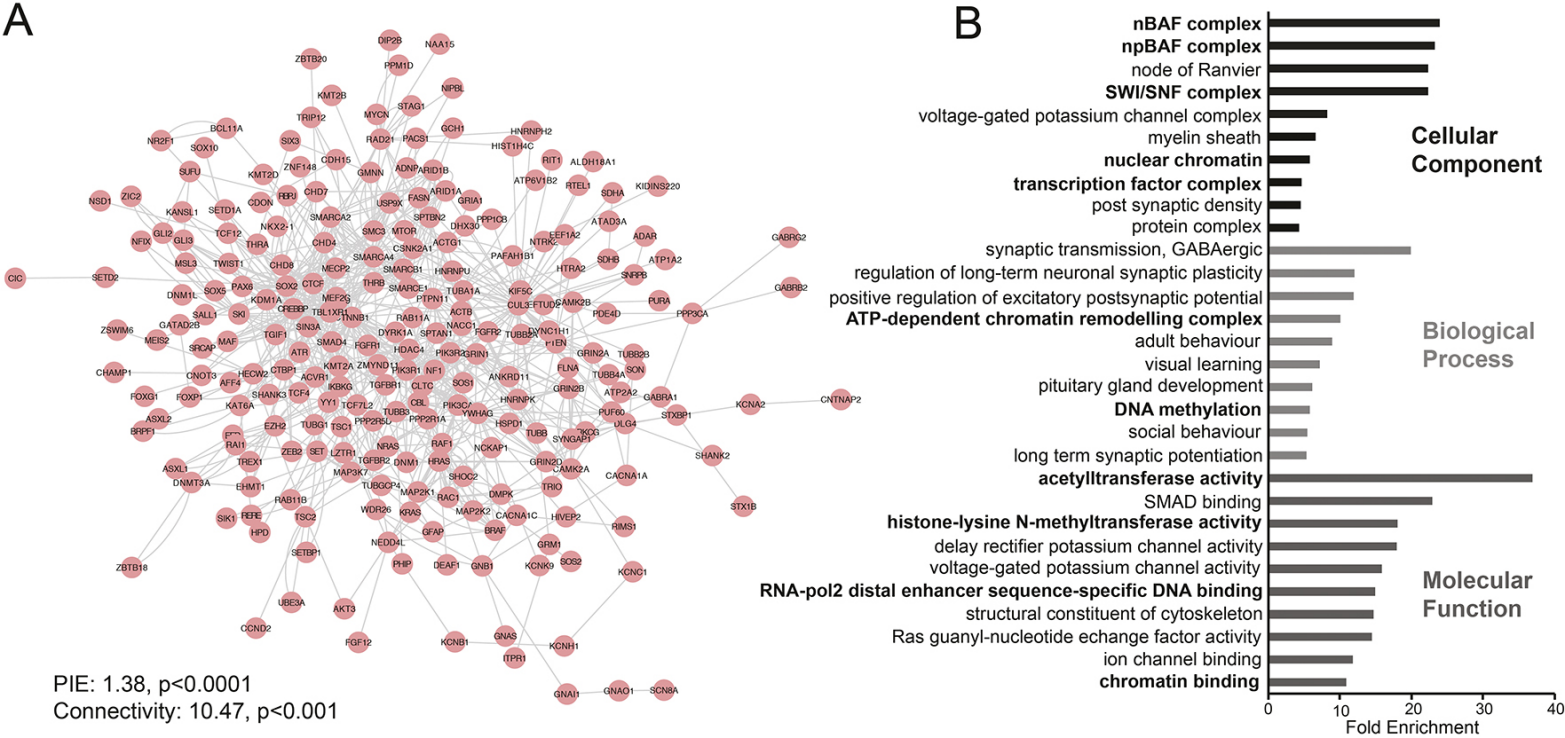
**DIGs are highly cohesive and enriched for the SWI/SNF chromatin remodelling complex.** (A) Protein interaction network of 339 DIGs obtained from sysID (sysid.cmbi.umcn.nl). 235 DIGs form a single network based on annotated protein–protein interactions in BioGrid small-scale studies and the Human Protein Reference Database. DIGs have significantly more interactions and connectivity than expected by random chance (PIE algorithm). (B) GO enrichment analysis for 339 DIGs. Top ten enriched terms with a Bonferroni corrected *P*-value <0.05 for each GO category are shown. Terms related to gene and chromatin regulation are shown in bold.

Considering that most ID cases are caused by dominant *de novo* mutations, we investigated whether known DIGs possess any common functionality. Using sysID (Kochinke et al., 2016) we retrieved a list of 339 known primary DIGs. Using data from BioGrid (Chatr-Aryamontri et al., 2017) and the Human Protein Reference Database (Keshava Prasad et al., 2009), we asked whether DIGs are involved in annotated protein–protein interactions (PPIs) with each other. Strikingly, 235 of the 339 genes form a single PPI network (Fig. 1A). Statistical analysis using the protein interaction enrichment (PIE) algorithm (Sama and Huynen, 2010) shows that the number of PPIs between DIGs is almost 40% more than would be expected with a random set of proteins with an equal number of known PPIs (PIE value=1.38, *P*<0.0001). Also, the connectivity of the DIG PPI network is nearly 11-fold higher than expected (*P*<0.001). This implies that DIGs are highly cohesive and may operate in similar biological processes or pathways.

To further investigate the potential overlapping function of DIGs, we performed gene ontology (GO) enrichment analysis. Enriched terms related primarily to neuronal components and functions (Fig. 1B, non-bold terms), or chromatin regulation (Fig. 1B, bold terms). The most enriched GO terms for cellular components were related specifically to the SWI/SNF chromatin remodelling complex, and one of the most enriched terms for biological processes was ‘ATP-dependent chromatin remodelling complex’. This demonstrates that disruption of chromatin regulation is a major factor in the aetiology of ID and identifies the SWI/SNF complex as the most over-represented protein complex associated with ID. The SWI/SNF complex is known to be essential for differentiation and cell-type specification in neuronal progenitors (Sokpor et al., 2017). However, individuals with SWI/SNF mutations do not appear to have major defects in differentiation or tissue specification, suggesting that SWI/SNF likely has important roles in post-mitotic neurons. Aside from the Baf53b subunit, which is exclusively expressed in post-mitotic neurons in mice (Lessard et al., 2007), little is known about the role of individual SWI/SNF subunits in differentiated neurons. Therefore, we set out to systematically investigate the function of SWI/SNF components in post-mitotic memory-forming neurons in *Drosophila*.

### Establishment of tools for investigation of SWI/SNF components in *Drosophila* memory-forming neurons

**Fig. 2.**
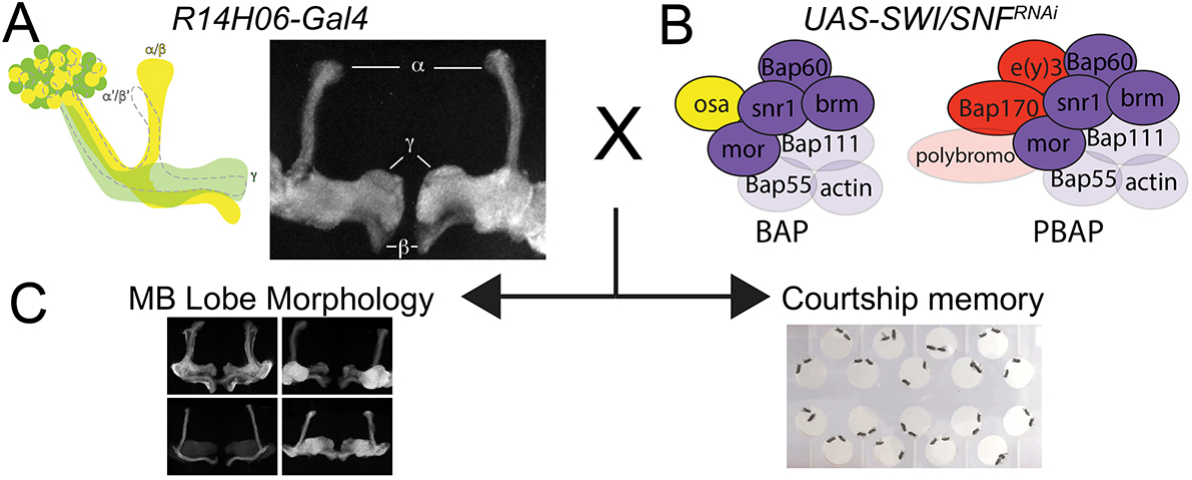
**Experimental strategy for investigating the function of individual SWI/SNF components in memory-forming neurons of the *Drosophila* MB.** (A,B) The MB-specific Gal4 driver *R14H06-Gal4* (A) was used to express *UAS-RNAi* lines targeting seven different components of the *Drosophila* SWI/SNF complex (B). (C) SWI/SNF knockdown flies and controls were examined for defects in MB morphology and courtship memory. A schematic diagram and confocal projection showing the expression domain of *R14H06-Gal4* is shown in A, and a full brain confocal stack is available in Movie 1. B shows a schematic representation of the BAP and PBAP conformations of the SWI/SNF complex. Purple, core and ATPase modules; yellow, BAP-specific subunits; red, PBAP-specific subunits. Subunits with validated RNAi lines used in this study are indicated with solid colour; other subunits are indicated by transparent colour.

Because null mutations in most SWI/SNF complex components cause embryonic lethality, we chose to specifically target memory-forming neurons of the *Drosophila* MB using the UAS/Gal4 binary expression system in combination with flies containing Gal4-inducible UAS-RNAi transgenes (see Materials and Methods). MB specificity was achieved using the Gal4 driver *R14H06-Gal4* from the Janelia FlyLight collection (Jenett et al., 2012), which is primarily expressed in post-mitotic cells contributing to the γ and α/β lobes of the adult MB (Fig. 2A; Movie 1) (Jones et al., 2018).

We aimed to test two RNAi constructs targeting different regions of the mRNA for ten established *Drosophila* SWI/SNF subunits: Brm, Bap60, Snr1, Bap111, Mor, Osa, E(y)3, Bap170, Bap55 and Polybromo (Fig. 2B). Although two RNAi lines were available for Polybromo, one of them [Vienna *Drosophil*a Resource Center (VDRC) stock number 108618] did not survive well under normal culture conditions. For the remaining genes, we initially tested RNAi efficiency by measuring percentage survival of adult progeny when RNAi transgenes were expressed ubiquitously using *Actin-Gal4* (Table S1). Of 19 RNAi lines tested, 15 caused near-complete lethality (survival <5%). Two RNAi lines were excluded (*UAS-Bap55^31708^* and *UAS-brm^34520^*), because they did not decrease the rate of survival compared to that of controls. An additional two RNAi lines showed an intermediate percentage survival that was still statistically less than that of controls: 53% for *UAS-Snr1^12644^* and 17% for *UAS-Bap60^33954^* (Table S1). For most RNAi transgenes, we performed additional validation of RNAi efficiency by quantitative PCR (qPCR) upon ubiquitous knockdown in whole larvae. We observed a decrease in mRNA using *UAS-brm^37720^* and *UAS-brm^31712^* (Table S1), each of which cause lethality in combination with the ubiquitous *Actin-Gal4* driver line (Mainland et al., 2017). In contrast, the *UAS-brm^34520^* RNAi line, which caused no reduction in survival in combination with *Actin-Gal4*, also caused no reduction in mRNA levels compared to controls (Table S1). This suggests that the lethality assay is a good proxy towards judging the efficiency of the RNAi lines used in this study. All other RNAi lines that were tested by qPCR showed a significant reduction in mRNA levels compared to controls, with the exception of those targeting *Bap111*. Despite inducing lethality upon expression with *Actin-Gal4*, *UAS-Bap111^35242^* showed increased mRNA levels by qPCR, while *UAS-Bap111^26218^* showed no change in mRNA levels. These results were confirmed at the protein level by western blotting (Fig. S1) and, therefore, Bap111 was excluded from further analysis. Here, we focus our investigation on the seven SWI/SNF subunits for which we had two validated RNAi lines. This included three components of the SWI/SNF core module (Bap60, Snr1 and Mor), one component of the ATPase module (Brm), the BAP-specific subunit Osa, and the PBAP-specific subunits E(y)3 and Bap170 (Fig. 2B). For each RNAi line, we looked for defects in MB morphology and memory upon expression with *R14H06-Gal4* (Fig. 2C).

### SWI/SNF components in the regulation of MB morphogenesis

The *R14H06-Gal4* driver is expressed in post-mitotic neurons of the MBγ and MBα/β lobes (Fig. 2A). MBγ neurons begin to arise in the early larval stage, whereas α/β neurons arise during pupal development (Lee et al., 1999). It was possible that SWI/SNF knockdown might regulate any post-mitotic processes affecting MB morphology, such as axonogenesis. Therefore, SWI/SNF knockdown MBs were examined for gross morphological defects using confocal microscopy on dissected whole-mount adult brains. This revealed five distinct phenotypic classes that were observed at various frequencies across the 348 adult fly brains that were imaged as part of this experiment. These phenotypes included: (1) missing α and β lobes, (2) β-lobe fibres crossing the midline, (3) stunted γ lobes, (4) extra-dorsal projections and (5) faded γ lobes. Phenotypes were qualitatively assessed for severity and compared statistically to relevant control genotypes (see Materials and Methods).

The first two phenotypic classes, missing α and β lobes, and β-lobe midline crossing, were observed across many knockdown genotypes and controls at a low frequency. The appearance of missing α and β lobes was very rare, occurring in 2.7% of control brains and 3.0% of knockdown brains. In addition, there was no significant difference between knockdown and control genotypes for any RNAi line (Fig. S2). The β-lobe crossing phenotype was observed more frequently, with 12.6% of control brains and 18.7% of knockdown brains showing a phenotype, usually of mild or moderate severity (Fig. S3). However, there were no cases in which two RNAi lines targeting the same gene caused a significant increase in the occurrence of this phenotype. Sporadic appearance of missing lobes and β-lobe crossing phenotypes were previously reported in a study looking at variation in MB morphology across different genetic backgrounds (Zwarts et al., 2015). Therefore, taken together with our findings, it seems unlikely that these phenotypes are specifically related to SWI/SNF RNAi knockdown.

**Fig. 3.**
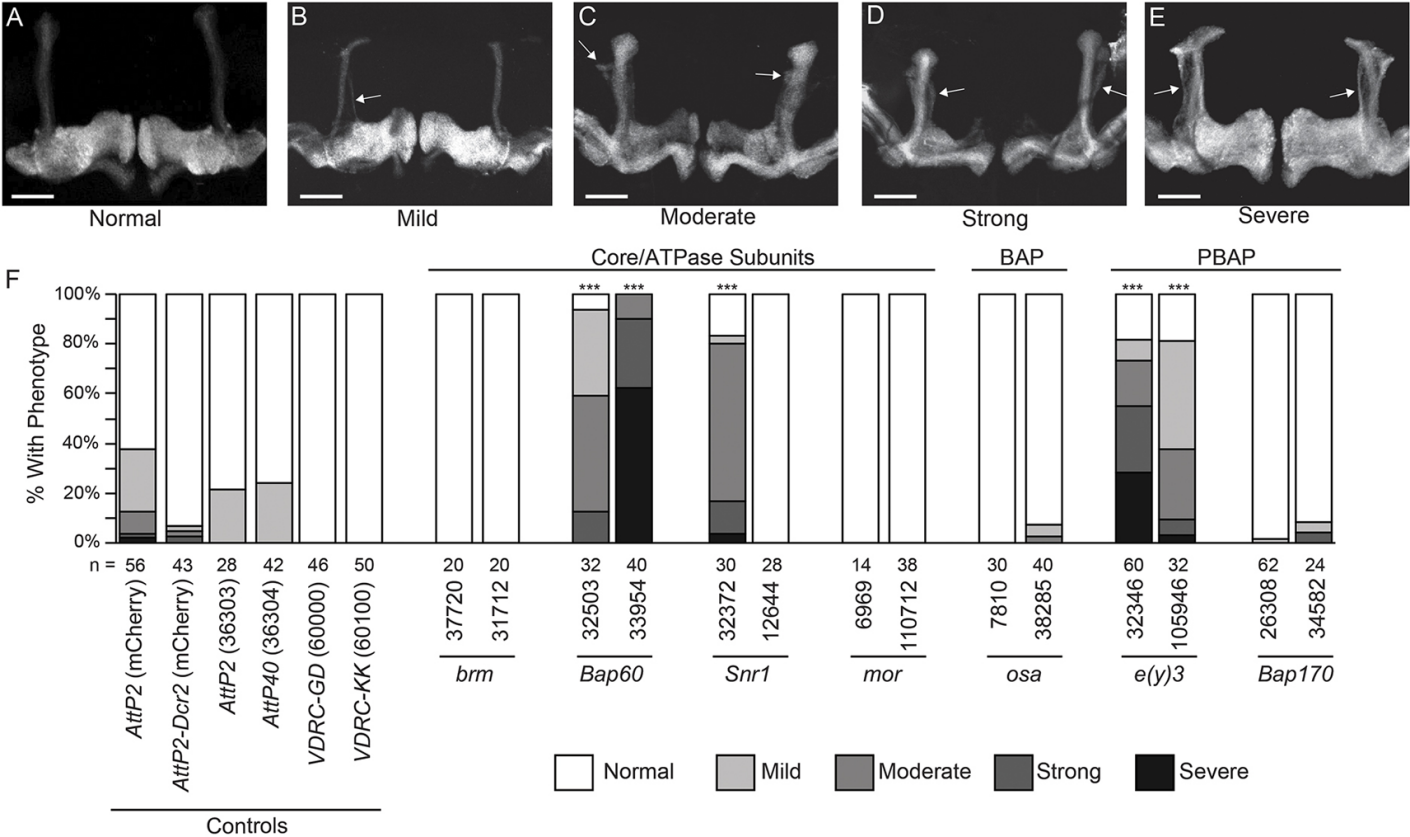
**Quantification of extra-dorsal projections in SWI/SNF knockdown MBs.** (A-E) The appearance of extra-dorsal projections was qualitatively classified into four categories to account for the observed variation in phenotype severity. Confocal projections show representative images for normal MB morphology (A), as well as the mild (B), moderate (C), strong (D) and severe (E) extra-dorsal projection phenotypes. Scale bars: 50 μm. Arrows indicate the location of extra-dorsal projections. (F) Bar chart showing the total percentage of brains exhibiting normal (white), mild (light grey), moderate (mid grey), strong (dark grey) and severe (black) extra-dorsal projections. The total number of MBs analysed for each genotype is indicated below the bars. ***P<0.001; Fisher's exact test, Bonferroni–Dunn test for multiple comparisons.

The next two observed phenotypic classes, stunted γ lobes and extra-dorsal projections, appeared to coincide in certain knockdown genotypes at a high penetrance. RNAi lines targeting *Bap60*, *Snr1* and *e(y)3* resulted in a near complete penetrance of the extra-dorsal projection phenotype, which was significantly greater than that seen in controls (Fig. 3). This phenotype was consistent between two RNAi lines for *Bap60* and *e(y)3*, suggesting that it is not likely due to off-target RNAi effects. For *Snr1*, the phenotype was not consistent between two RNAi lines tested, with *UAS-Snr1^12644^* causing no phenotype. This discrepancy is likely a result of the weaker knockdown efficiency observed with *UAS-Snr1^12644^*, which showed a 53% survival rate in our *Actin-Gal4*-induced lethality assay, compared to 4% for *UAS-Snr1^32372^* (Table S1). For *Bap60* and *Snr1* RNAi lines, the appearance of a severe extra-dorsal projection phenotype corresponded with the presence of stunted γ lobes, indicating that these two phenotypes could be linked (Fig. S4). Overall, these findings suggest that Bap60, Snr1 and E(y)3 may regulate specific aspects of MB morphogenesis.

### Specific SWI/SNF components are required for axon pruning during MBγ neuron remodelling

**Fig. 4.**
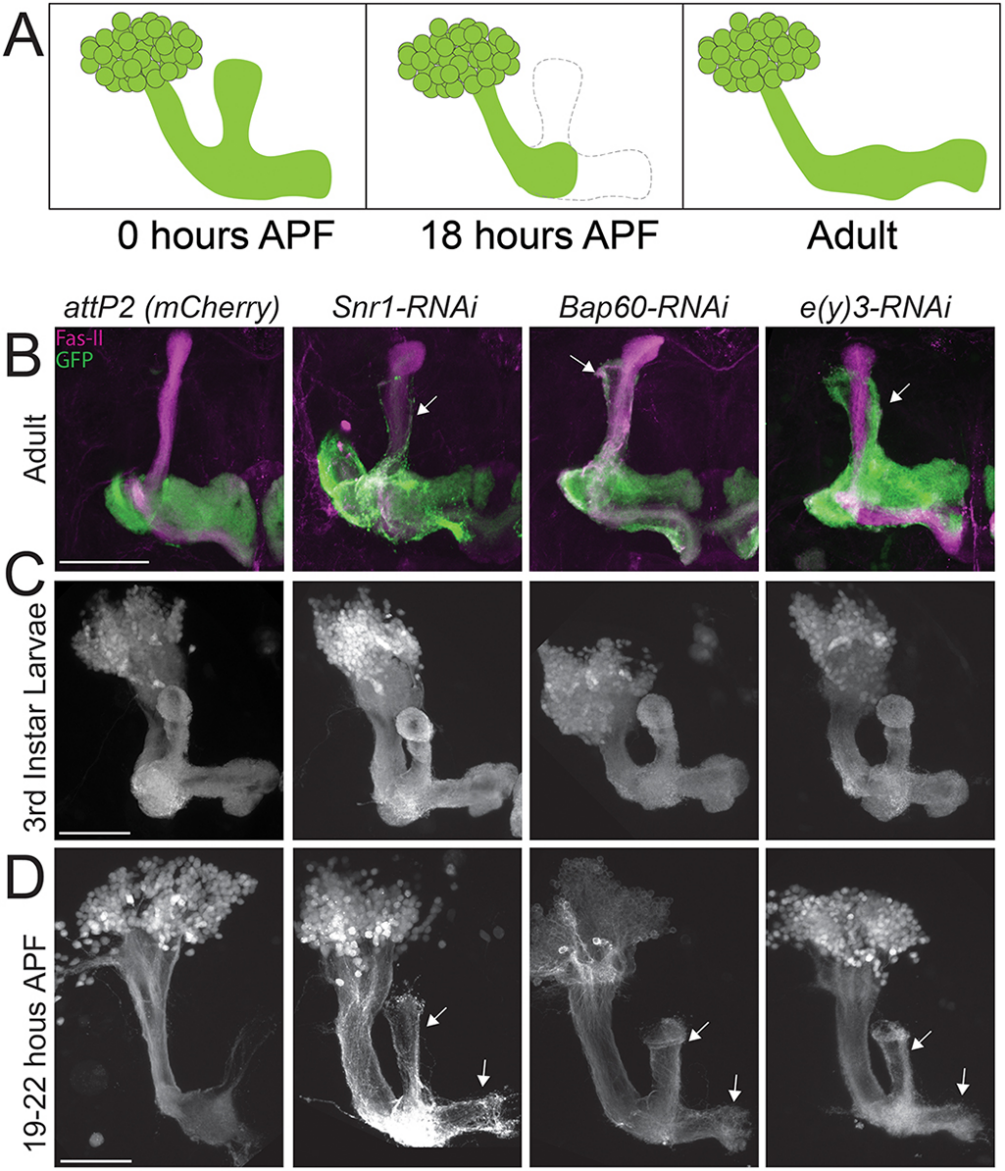
**Some SWI/SNF components are required for MBγ neuron remodelling.** (A) Schematic diagram of MBγ neuron remodelling. APF, after pupae formation. The dashed line indicates the part of the MBγ lobe that is pruned. (B-D) Confocal projections showing MB neurons labelled with *R14H06-Gal4* and *UAS-mCD8::GFP*. Controls expressing an RNAi against mCherry were compared to SWI/SNF knockdown RNAi lines for Bap60 (*UAS-Bap60^32503^*), Snr1 (*UAS-Snr1^32372^*) and E(y)3 [*UAS-e(y)3^32346^*]. Images were obtained for adults (B), third-instar larvae (C) and early pupae (D). FasII was labelled by immunohistochemistry. Scale bars: 50 µm. Arrows indicate the location of unpruned MBγ axons. For each genotype and developmental stage, we imaged a minimum of ten brains. Larval (C) and pupal (D) phenotypes were 100% penetrant. The penetrance of adult phenotypes is quantified in Fig. 3.

Previous studies have shown that extra-dorsal projections can arise due to defects in MBγ neuron remodelling that occur during pupal morphogenesis (Boulanger et al., 2011; Lai et al., 2016; Lee et al., 2000). During the larval stages of development, MBγ neurons project both dorsally and medially. During the first 18 h of pupal development, the dorsal and medial projections are pruned back to the peduncle. This is followed by re-extension of the γ neurons medially, but not dorsally, to form the adult γ lobe (Lee et al., 1999) (Fig. 4A). The extra-dorsal projections that we observed in adult brains with Bap60, Snr1 and E(y)3 knockdown (Fig. 4B) might have resulted from defects in remodelling of MBγ neurons, or from aberrantly formed α/β neurons. We performed RNAi knockdown using two previously characterized MBγ-specific split-Gal4 lines: *MB607B-Gal4*, expressed in the γd subset of MB neurons, and *MB009B-Gal4*, expressed in the γd and γmain neurons, which form the bulk of the γ lobe (Aso et al., 2014). Knockdown of Bap60 and E(y)3 using *MB607B-Gal4* and *MB009B-Gal4* resulted in the appearance of extra-dorsal projections in adult flies (Fig. S5), demonstrating that this morphological defect results from defects in γ neuron morphology and not from misrouting of α/β neurons. Some Snr1 knockdown flies showed extra-dorsal projections with *MB607B-Gal4* and *MB009B-Gal4*; however, the proportion of flies with a phenotype was not different from controls. Interestingly, all Snr1 knockdown MBs that did not have extra-dorsal projections showed a clear reduction in the volume of axons (Fig. S5). This is consistent with the severe ‘stunted γ’ phenotype seen with *R14H06-Gal4* *Snr-1* knockdown (Fig. S4), indicating that Snr1 may have a dual role in regulating MBγ neuron morphology and survival.

Next, we investigated MB γ-lobe structure at the larval and pupal stages to see whether extra-dorsal projections resulted from defects in pruning, or aberrant re-extension of γ neurons. Knockdown of Bap60, Snr1 and E(y)3 caused no notable defects in larval MB morphology, suggesting that axon pathfinding can occur normally in knockdown flies (Fig. 4C). In pupae, MBγ neuron pruning was observed at 19-22 h after pupation in controls, as expected (Fig. 4D). In contrast, pupae with knockdown of Bap60, Snr1 and E(y)3 did not show MBγ axon pruning (Fig. 4D). This defect was 100% penetrant for all three SWI/SNF subunits and was specific for axons, as pruning of MB dendrites was not affected (Fig. 4C,D). This verified that the extra-dorsal projection phenotype observed in adult flies is the result of defects in MBγ neuron pruning during pupal morphogenesis.

### Knockdown of Osa and Brm causes age-dependent loss of MBγ axons

**Fig. 5.**
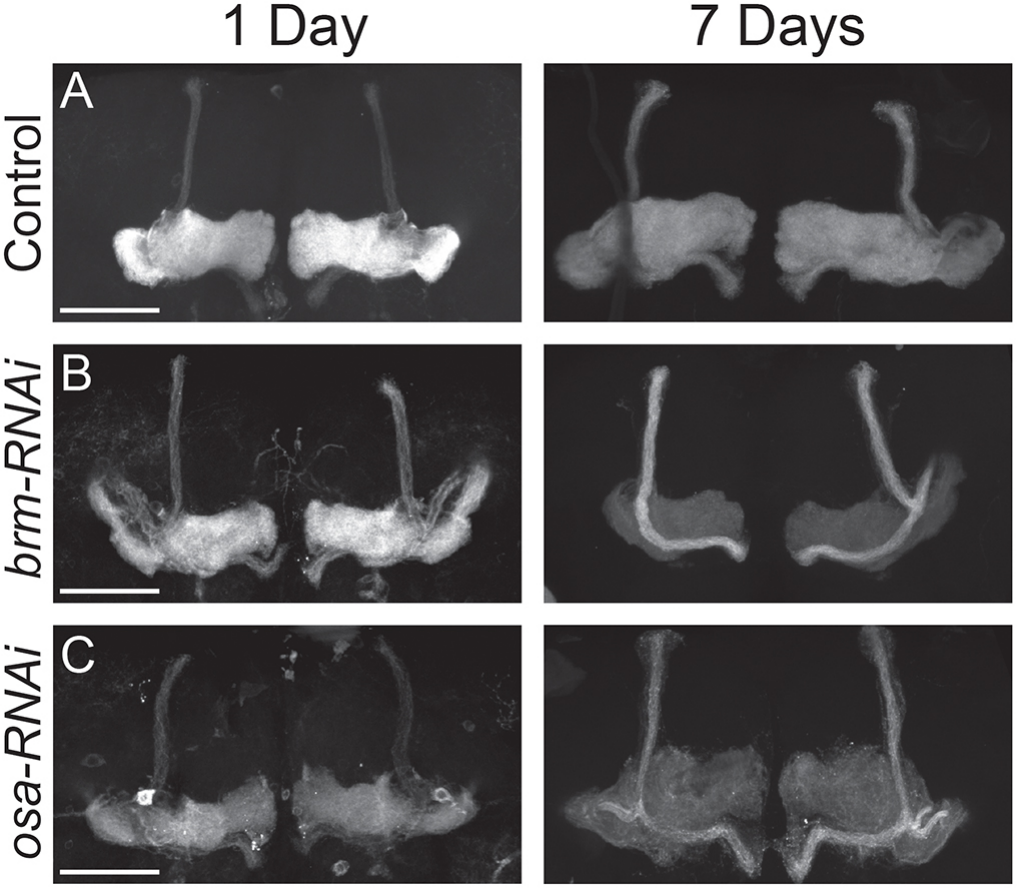
**Some SWI/SNF components are required for MBγ axon survival during ageing.** (A-C) Confocal projections showing MB neurons labelled with *R14H06-Gal4* and *UAS-mCD8::GFP* at 1 and 7 days after eclosion. Controls expressing an mCherry RNAi (A) are compared to flies expressing RNAi constructs targeting *brm* (*UAS-brm^31712^*) (B) and *osa* (*UAS-osa^7810^*) (C). Phenotypes shown were highly consistent in at least ten individual brains for each genotype and time point. Scale bars: 50 µm.

The final observed morphological phenotype, described as faded γ lobes, is characterized by normal MB morphology, with a shift in the intensity of fluorescent labelling by GFP. *R14H06-GAL4* is specifically expressed in the α/β and γ neurons of the MB (Jenett et al., 2012). In controls, GFP expression is strongest within the γ lobe and weakest within the α/β lobes (Figs 2A and 5A). For some genotypes, SWI/SNF knockdown caused the appearance of faint γ lobes that were otherwise morphologically normal. Initial attempts at quantification of this phenotype produced a high level of variability. However, the phenotype was most strongly and consistently observed upon RNAi knockdown of Brm and Osa. Because our initial screen included flies between 1 and 7 days of age, we reasoned that the high variability observed in the γ-fade phenotype may be due to the variable age of the flies tested. Therefore, we tested the effects of age in Brm and Osa knockdown flies. At 1 day, knockdown flies looked similar to controls. However, at 7 days, γ-lobe fluorescence was clearly and consistently reduced in Brm and Osa knockdown MBs (Fig. 5A-C). Thus, some SWI/SNF components are required to maintain the survival of MBγ axons during ageing.

### MB-specific SWI/SNF knockdown causes memory defects

Next, we asked whether SWI/SNF knockdown would affect the function of MB neurons in memory. To test MB-specific SWI/SNF knockdown flies for defects in learning and memory, we used a classic assay called courtship conditioning (Koemans et al., 2017; Siegel and Hall, 1979). This assay involves experience-based modification of male courtship behaviour towards female flies. Naïve males court females at a high frequency, but pre-mated females are non-receptive and will reject male courtship attempts. After rejection by a non-receptive female, male flies exhibit a learned reduction of courtship behaviour. Short-term memory (1 h after rejection) can be induced by a 1-h period of sexual rejection, while long-term memory (24 h after rejection) can be induced by a 7-h training period. Analysis of short- and long-term courtship memory was performed for six different control genotypes that represent different genetic backgrounds associated with different RNAi lines (see Materials and Methods). Each of the six control genotypes demonstrated a significant reduction in courtship index (CI; proportion of time spent courting) relative to naïve flies for both short- and long-term memory (Figs S6 and S7). The average control learning index (LI), which is the proportional reduction in CI due to training, was 0.42±0.0096 for short-term memory and 0.24±0.017 for long-term memory (Figs S6 and S7), which is consistent with previous studies (Keleman et al., 2012; Kramer et al., 2011). As such, courtship conditioning is effective for eliciting short- and long-term memory across a variety of different control strains that represent different genetic backgrounds of the MB-specific SWI/SNF RNAi knockdown flies.

**Fig. 6.**
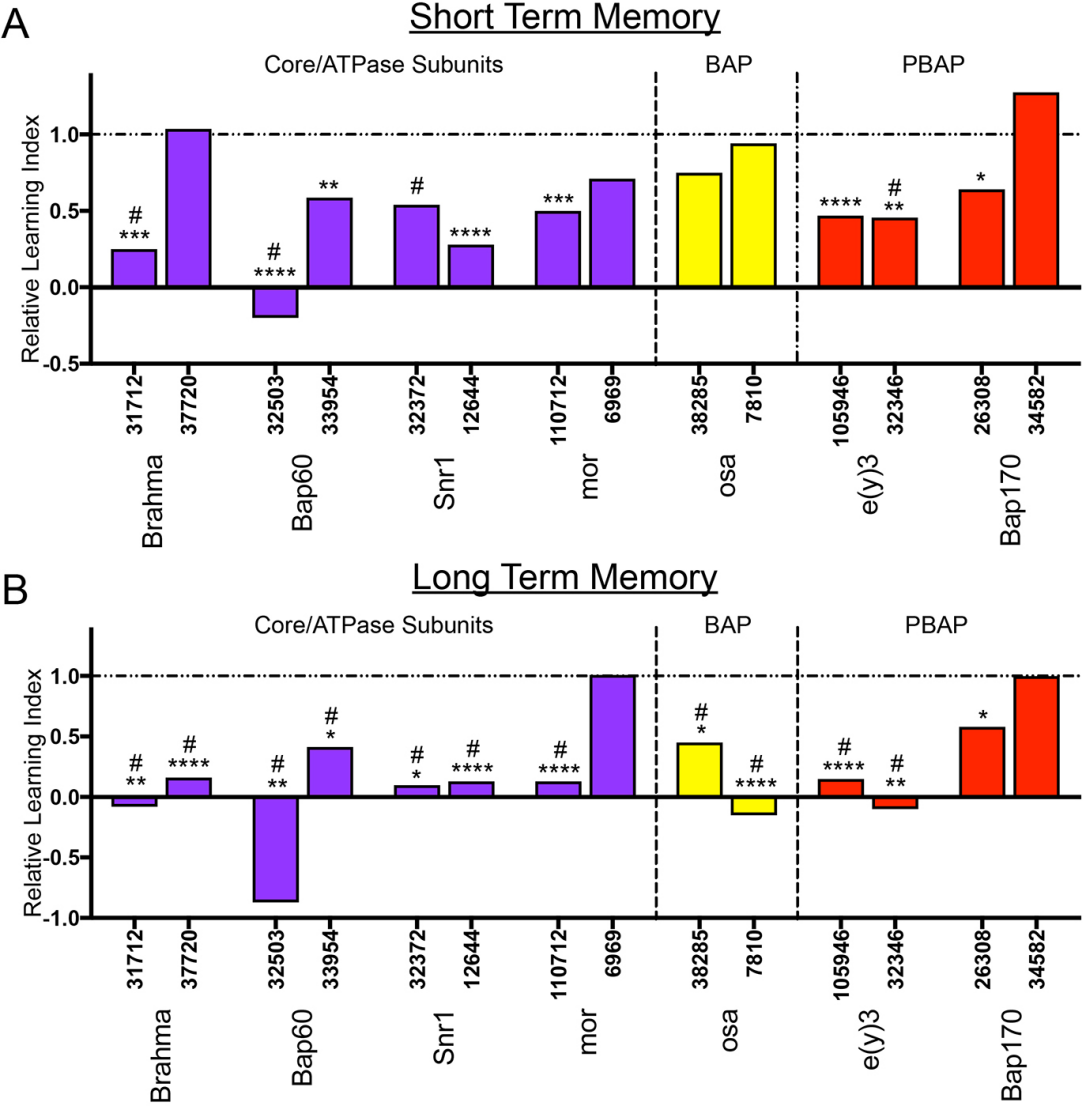
**SWI/SNF complex components are required in the MB for short- and long-term courtship memory.** (A,B) The relative learning index (LI) of SWI/SNF RNAi knockdown flies compared to their appropriate genetic background control (see Materials and Methods) for short-term memory (A) and long-term memory (B). Purple bars represent SWI/SNF subunits from the core and ATPase modules; yellow bars represent BAP-specific subunits; and red bars represent PBAP-specific subunits. # indicates a memory defect indicated by no significant reduction in courtship index (CI) in naïve flies compared to trained flies for that genotype (Kruskal–Wallis test). Raw CI and LI data are available in Figs S6 and S7. *P<0.05, **P<0.01, ***P<0.001, ****P<0.0001; randomization test, 10,000 bootstrap replicates.

We examined short- and long-term memory in MB-specific SWI/SNF knockdown flies. Fig. 6 shows the relative LI for each RNAi line compared to the appropriate genetic background control (see Materials and Methods and Table S2). The raw courtship data and LIs are shown in Figs S6 and S7. Memory defects were widespread among the different SWI/SNF subunits (Fig. 6). Generally, long-term memory was more affected, with 86% of RNAi lines inducing a defect (Fig. 6B), compared to 64% for short-term memory (Fig. 6A). For Brm, Bap60, Snr1, Osa and E(y)3, we observed memory defects that were consistent between two RNAi lines. Only Mor and Bap170 showed inconsistencies in memory phenotypes between the two RNAi lines, with only one RNAi line inducing memory loss. Interestingly, while most RNAi lines affect both short- and long-term memory, only the BAP-specific subunit Osa shows a specific defect in long-term memory. These data suggest that the SWI/SNF complex is important for the normal functioning of MB neurons in memory formation, and that the BAP complex, which is defined by the presence of Osa, might have a more specific role in the regulation of long-term memory.

## DISCUSSION

Our knowledge of genetic causes of ID is vast. Here, we show that DIGs are highly connected and enriched for functions related to neuronal signalling and chromatin regulation (Fig. 1). This suggests that many different ID syndromes, which are defined by mutations in different genes, might result from disruption of common cellular and molecular processes. Understanding these common processes represents a significant challenge. Here, we focus on the SWI/SNF complex, which is the most enriched cellular component among DIGs (Fig. 1B). The role of this complex in cell-type specification and differentiation is well understood; however, its function in post-mitotic neurons has not been extensively investigated (Son and Crabtree, 2014). In a screen targeting memory-forming neurons of the *Drosophila* MB, we identified a novel role for several individual components of this complex in post-mitotic neuronal processes, including neuron remodelling, survival and memory formation (Figs 3-6). Interestingly, different components of the complex seem to have distinct roles in post-mitotic neurons. Bap60, Snr1 and E(y)3 are required for morphological remodelling of MBγ axons during metamorphosis, while Osa and Brm are required for maintenance of MBγ axon survival during ageing (Figs 4 and 5). Most SWI/SNF components are required for both short- and long-term memory, with the exception of the BAP-specific subunit Osa, which only affects long-term memory (Fig. 6). This study has revealed new roles for several SWI/SNF components in the biology of memory-forming neurons and provides a broad phenotypic analysis that will serve as a basis for further investigation into the underlying gene regulatory mechanisms.

### SWI/SNF components in the regulation MBγ neuron remodelling

We show here that the PBAP-specific SWI/SNF subunit E(y)3 and the core subunits Bap60 and Snr1 are required for the pruning back of MBγ axons during the early stages of pupal metamorphosis (Fig. 4). Brm, the SWI/SNF ATPase subunit, has previously been implicated in the pruning of multidendritic neurons lining the body wall in the *Drosophila* larvae (Kirilly et al., 2011). Using a dominant-negative *brm* transgene, Kirilly et al. also identified a role for Brm in MBγ neuron pruning in pupae. We did not observe a MBγ remodelling defect upon *brm* RNAi knockdown. This suggests that RNAi did not sufficiently reduce Brm protein levels in order to induce a phenotype. Indeed, we see that *brm* RNAi lines reduce mRNA levels to about 50%, suggesting that some protein is likely still produced. The dominant-negative *brm* may be more efficient in silencing SWI/SNF complex activity to induce remodelling defects.

It is well established that ecdysone signalling is critical for MBγ neuron remodelling (Lee et al., 2000). Ecdysone receptor B1 (EcR-B1; also known as EcR) expression is induced in the MB in the late third instar, initiating the expression of downstream transcription factors that are thought to control gene expression programs required for pruning. Kirilly et al. showed that Brm functions downstream of EcR-B1 and that it may regulate pruning by inducing expression of the ecdysone-responsive transcription factor, Sox14 (Kirilly et al., 2011). Consistent with this, we found EcR-B1 levels to be unaltered after knockdown of Bap60 and E(y)3 (Fig. S8). Although it is clear that SWI/SNF components regulate pruning downstream of EcR-B1, the specific mechanisms remain to be determined and many questions still remain. Why is it that some, but not all, SWI/SNF components are required for MBγ remodelling? Perhaps some SWI/SNF components mediate critical protein–protein interactions with transcription factors that are specifically required for remodelling. A recent study identified several DNA-binding proteins that are required for MBγ pruning (Alyagor et al., 2018), which are interesting candidates that might interact with SWI/SNF components. It is also possible that only certain configurations of the SWI/SNF complex are involved in pruning. For example, we see that the PBAP component E(y)3 is essential for pruning, whereas the BAP-specific subunit Osa is not (Fig. 3). Finally, different subunits might have different specificities because of their unique effects on the modular assembly of the different SWI/SNF complex configurations (Mashtalir et al., 2018).

The JNK signalling pathway has also been implicated in pruning of MBγ axons by inhibiting cell adhesion though a transcription-independent mechanism that operates in parallel to ecdysone signalling (Bornstein et al., 2015). Loss of the JNK orthologue *basket* specifically affects MBγ axon pruning and not dendrite pruning. Interestingly, we also observe an axon-specific effect on pruning upon knockdown of SWI/SNF components (Fig. 4). Therefore, it would be interesting to see whether SWI/SNF somehow interacts with the JNK signalling pathway to mediate axon-specific pruning of MBγ neurons.

### SWI/SNF components prevent age-dependent loss of MBγ axons

RNAi knockdown of some SWI/SNF components, including Brm and the BAP-specific subunit Osa, caused an age-dependent loss of MB axons (Fig. 5). This suggests that, in some contexts, loss of the SWI/SNF complex may be associated with axon degeneration or neuronal cell death. There is currently no indication that SWI/SNF-associated ID disorders have a neurodegenerative component (Mari et al., 2015; Santen et al., 2013); however, this cannot be ruled out as ID is typically diagnosed at a very young age. Interestingly, mutations in the human SWI/SNF gene *SS18L1* have been implicated in amyotrophic lateral sclerosis (Chesi et al., 2013), a neurodegenerative disorder characterized by loss of motor neurons. Therefore, our analysis, targeting post-mitotic neurons, may have revealed an unappreciated role for the SWI/SNF complex in regulating age-dependent neuronal survival. While we have demonstrated an age-dependent effect for Brm and Osa, it is possible that other SWI/SNF components are also important for MBγ axon survival. For example, knockdown of both Snr1 and Bap60 in our initial phenotypic screen caused a strong ‘stunted γ’ phenotype (Fig. S4), and Snr1 knockdown caused a clear reduction in axons with two MBγ-specific split-Gal4 lines (Fig. S5). These phenotypes might also be caused by death or degeneration of MBγ axons. Although Snr1- and Bap60-associated axon loss does not appear to be age dependent, it does suggest a broad role for different SWI/SNF components in MBγ axon survival. Further studies are required to determine the mechanisms and the full extent of different SWI/SNF components in maintaining axon survival during ageing.

### Different roles for SWI/SNF components in memory

We showed that most SWI/SNF subunits are required in the MB for memory (Fig. 6). However, what is the underlying cause of these memory phenotypes? Our study suggests that individual SWI/SNF components have different roles in MB neurons depending on the developmental context. The SWI/SNF components tested here had no effect on the early formation of the MB in the larval stages (Fig. 4C). In contrast, the complex is important for MBγ remodelling during early pupal development (Fig. 4D). This dramatic remodelling of the physical structure of the MB in the pupae is known to be required for normal short-term courtship memory in adult flies, but not for long-term memory (Redt-Clouet et al., 2012). For some SWI/SNF components, loss of short-term memory correlates with a strong defect in MBγ remodelling. However, we observed several SWI/SNF knockdown genotypes with memory defects that occurred in the absence of remodelling defects, suggesting that SWI/SNF complexes are also important in adult MB neurons. Indeed, we observed an adult-specific role for some SWI/SNF components in age-dependent neuron survival (Fig. 5). In a parallel study, we showed that adult-specific Bap60 MB knockdown causes memory defects and that Bap60 was critical for MB-specific gene expression in early juvenile adults (Nixon et al., 2018), when the MB is thought to undergo experience-dependent plasticity that shapes adult circuitry required for memory (Barth and Heisenberg, 1997). Thus, different SWI/SNF subunits appear to affect multiple aspects of MB neurobiology during the lifetime of a fly. Disruption of any one of these different factors might impact the ability of MB neurons to encode memories.

Another interesting possibility is that SWI/SNF complexes might impact memory through regulation of neuron-activity-induced genes that are required for normal long-term memory, but not short-term memory (Alberini and Kandel, 2014). We found that the BAP-specific SWI/SNF component Osa is required for long-term memory, but not short-term memory (Fig. 6). Osa was the only SWI/SNF component that was required for only one type of memory. All other RNAi lines affected both short- and long-term memory, or did not affect memory at all. This raises the possibility that the BAP complex is important for long-term memory processes such as neuron-activity-induced gene expression. Interestingly, the mammalian SWI/SNF complex mediates enhancer selection in fibroblasts in cooperation with FOS (Vierbuchen et al., 2017), a critical neuron-activity-induced transcription factor involved in memory (Gallo et al., 2018). It is not known whether mammalian SWI/SNF interacts with FOS in neurons. Nevertheless, our study provides a basis to further investigate the potential role of SWI/SNF in the context of *Drosophila* memory-induced gene regulation, and identifies the BAP-specific subunit Osa as an excellent candidate that is required for long-term memory, but not short-term memory.

## MATERIALS AND METHODS

### Network and GO analysis of DIGs

Network analysis of DIGs was performed as described (Kochinke et al., 2016), using annotated interactions from BioGrid 3.2.108 (released 1 January 2014) (Chatr-Aryamontri et al., 2017) and the Human Protein Reference Database (Keshava Prasad et al., 2009) (Release 9, 13 April 2010). The PIE score, connectivity and the associated *P*-values were calculated using the PIE algorithm (Sama and Huynen, 2010). GO analysis of DIGs was performed using DAVID, version 6.8 (Huang et al., 2009). The most enriched GO terms with a Bonferroni-corrected *P*-value <0.05 are shown.

### *Drosophila* stocks and crosses

Flies were reared at 70% humidity on a 12h-12 h light-dark cycle on standard cornmeal-agar medium. All of the RNAi lines and genetic background controls were obtained from either the Transgenic RNAi project (TRiP) (Perkins et al., 2015) [via the Bloomington *Drosophila* Stock Center (BDSC)] or the VDRC (Dietzl et al., 2007) (listed in Tables S1 and S2). The following additional stocks were obtained from the BDSC: *UAS-mCD8*::*GFP* (5137), *Pin^Yt^/CyO, UAS-mCD8::GFP* (5130), *UAS-Dcr-2* (24650), *R14H06-Gal4* (48667) and *Actin-Gal4/CyO* (25374). The MBγ split-Gal4 lines *MB607B-Gal4* and *MB009B-Gal4* were obtained from the FlyLight Collection at Janelia Research Campus (Ashburn, VA, USA) and have been described previously (Aso et al., 2014). Both driver lines are expressed specifically in different subsets of MBγ neurons in larvae, pupae and adult (Fig. S5).

To study the effects of SWI/SNF knockdown in post-mitotic memory-forming neurons, the UAS/Gal4 system was used for targeted RNAi using the MB-specific *R14H06-Gal4* driver line. The expression domain of *R14H06-Gal4* has been documented by the FlyLight project and is publicly available online (http://flweb.janelia.org) (Jenett et al., 2012). The driver shows a lack of expression in embryos and specific labelling of the MB in the larval and adult brain. Some cells in the ventral nerve chord are also labelled in the larval and adult stages, but these cells clearly have a much lower expression than the MB cells. We confirmed the *R14H06-Gal4* expression domain in adult brains (Fig. 2A; Movie 1) (Jones et al., 2018) and verified that Gal4 is specifically expressed in the MB in the pupal and larval stages (Fig. 4C,D; Fig. S9).

The RNAi constructs used in this study consist of both short and long hairpin RNA molecules. Dicer-1 is endogenously expressed in the fly, and this expression is effective for the processing of short hairpin RNAs (TRiP VALIUM20, VALIUM21 and VALIUM22 collections) (Ni et al., 2011; Perkins et al., 2015). However, long hairpin RNAi constructs require the co-expression of Dicer-2 to achieve optimal knockdown (TRiP VALIUM1 and VALIUM10 collections; VDRC GD and KK libraries) (Dietzl et al., 2007). Therefore, *UAS-Dicer-2* was co-expressed in the MB when long hairpin RNA lines were used for knockdown.

For analysis of MB morphology, RNAi lines (Table S2) were crossed to flies of the genotypes *UAS-mCD8::GFP/CyO; R14H06-GAL4/TM6* or *UAS-Dcr2/CyO; R14H06-GAL4, UAS-mCD8::GFP/TM6*, at 29°C. For courtship conditioning experiments, RNAi lines (Table S2) were crossed to *R14H06-Gal4* or *UAS-Dcr2/CyO; R14H06-GAL4/TM6*, at 25°C. For all knockdown experiments, control flies were generated by crossing the appropriate genetic background strain to the driver line (see Table S2 for controls used for each RNAi line). For knockdown experiments using TRiP RNAi lines, the corresponding controls were generated using either *attP2(36303)*, *attP40(36304)* or *attP2(mCherry)*. For knockdown experiments using RNAi lines obtained from the VDRC, controls were generated using *VDRC-GD(60000)* or *VDRC-KK(60100)*.

### Validation of RNAi lines by qPCR and lethality assay

As a simple phenotypic test to assess RNAi efficiency, we measured survival upon ubiquitous knockdown, with the knowledge that null mutations in most genes investigated in this study cause lethality. *Actin-Gal4/CyO* flies were crossed to *UAS-RNAi* flies for *brm*, *Bap60*, *Snr1*, *mor*, *Bap111*, *osa*, *e(y)3*, *Bap55*, *polybromo* and *Bap170* (Table S1). Percentage survival was calculated by comparing the number of progenies with straight wings to the number of flies with curly wings (not receiving *Actin-Gal4*). Validation of RNAi knockdown efficiency by qPCR was performed as described (Mainland et al., 2017), using polyA-purified RNA as a template and primers directed towards the 5′ side of the predicted RNAi-induced cleavage site. Western blotting was performed according to standard protocols using primary antibodies guinea pig anti-Bap111 (1:2000) (Chalkley et al., 2008) and mouse anti-β-tubulin [1:8000; Developmental Studies Hybridoma Bank (DSHB)], and horseradish-peroxidase-conjugated secondary antibodies goat anti-guinea pig (1:8000; Thermo Fisher Scientific) and goat anti-mouse (1:3000; Bio-Rad).

### Immunostaining and analysis of MB morphology

Brains were dissected in PBS and fixed with 4% paraformaldehyde for 45 min at room temperature, before mounting in Vectashield (Vector Laboratories). For immunohistochemistry, fixed brains were incubated overnight with the primary antibodies anti-FasII (1:25; DSHB, 1D1), anti-Brp (1:50; DSHB, nc82) and anti-EcR-B1 (1:25; DSHB, AD4.4), and the secondary antibody goat anti-mouse DyLight 594 (1:300). Images were acquired using a Zeiss LSM510 or Zeiss LSM800 confocal microscope. Confocal stacks were processed using ImageJ software (Schindelin et al., 2015) and Adobe Photoshop.

Gross MB morphology was assessed and qualitatively quantified by examining confocal stacks. Five distinct morphological variations were observed, including (1) missing α and β lobes, (2) β-lobe fibres crossing the midline, (3) extra-dorsal projections, (4) stunted γ lobes and (5) faded γ lobes. For missing lobes, each brain was scored as either missing a lobe or not. Due to variability in the severity, β-lobe crossing, extra-dorsal projections and stunted γ lobes were classified as ‘normal’, ‘mild’, ‘moderate’, ‘strong’ or ‘severe’, as indicated in Fig. 3 and Figs S3-S5. The β-lobe crossing phenotype was scored based on the width and density of GFP-labelled β-lobe fibres crossing the midline. Extra-dorsal projections were classified based on the number and thickness of dorsal projections. Stunted γ lobes were classified based on the relative size of the γ lobe.

### Courtship conditioning

Courtship conditioning was performed as described previously (Koemans et al., 2017). Male knockdown flies were collected at eclosion and raised in isolation for 5 days before pairing with a pre-mated female for 1 h, for short-term memory experiments, or 7 h, for long-term memory experiments. During training, pre-mated females reject male courtship attempts. Following training, males were placed in isolation for 1 h, for short-term memory experiments, or 24 h, for long-term memory experiments. Following isolation, both naïve and trained males were individually paired with a new pre-mated female in 18-well courtship chambers and courtship behaviour was recorded for 10 min. Experiments were conducted on at least three different days with a maximum of 18 male/female pairs per condition per day. For each of the males used in this study a CI was calculated through manual scoring of courtship behaviour by expert observers.

Statistically, loss of memory was identified using two complementary methods. Reduction of the mean CI of trained (CI_trained_) flies compared to naïve (CI_naïve_) flies of the same genotype was compared using a Kruskal–Wallis test followed by pairwise comparisons using Dunn's test. No significant reduction in the mean CI due to training (*P*>0.05) indicates a defect in memory. In some cases, a group of flies may show a significant reduction in CI due to training, but still show less capacity for memory compared to controls. For this comparison, an LI was calculated [LI=(CI_naïve_ –CI_trained_)/CI_naïve_]. LIs were compared between genotypes using a randomization test (Kamyshev et al., 1999) (10,000 bootstrap replicates) using a custom R script (Koemans et al., 2017), and the resulting *P*-values were corrected for multiple testing using the method of Bonferroni.

## Supplementary Material

Supplementary information
