# Supplementary material for: Individual components of the SWI/SNF chromatin remodelling complex have distinct roles in memory neurons of the Drosophila mushroom body
Source: Dis Model Mech. 2019 Mar 25;12(3):dmm037325. doi: 10.1242/dmm.037325 (PMC6451433; doi:10.1242/dmm.037325)
Supplement: Supplementary information [file dmm-12-037325-s1.pdf]

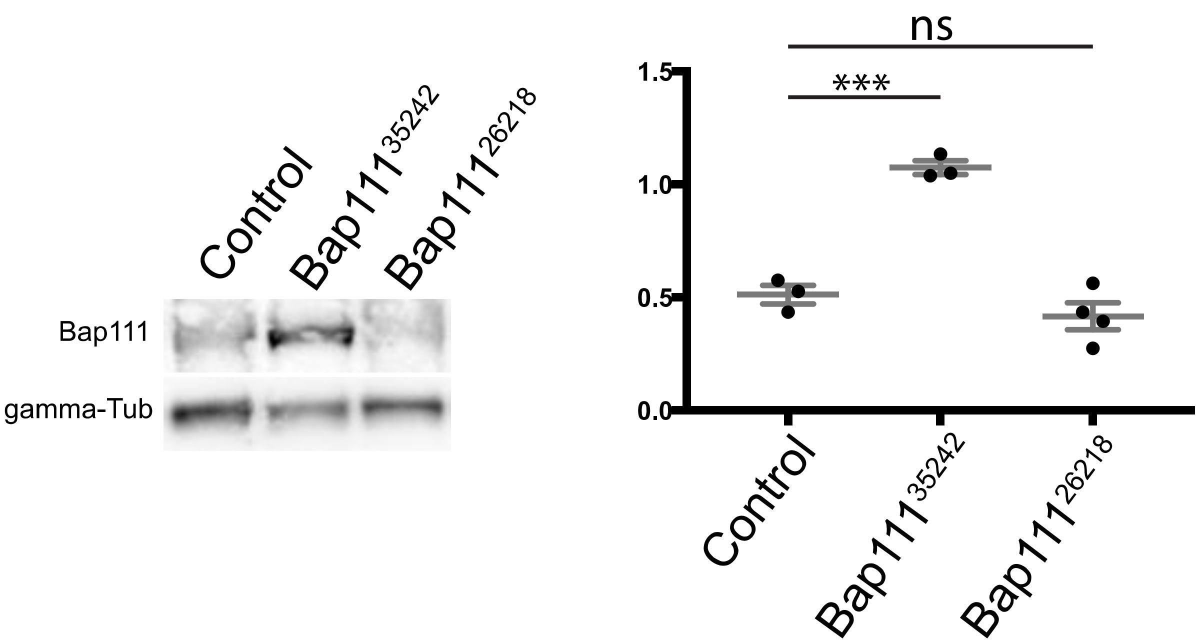

Fig. S1. Quantification of Bap111 expression in RNAi knockdown larvae by Western blot. Representative bands for Bap111 and the loading control gamma-tubulin (left). At least three biological replicates were quantified using image J (right). ****P*<0.001; ANOVA, Dunnett's test for multiple comparisons.

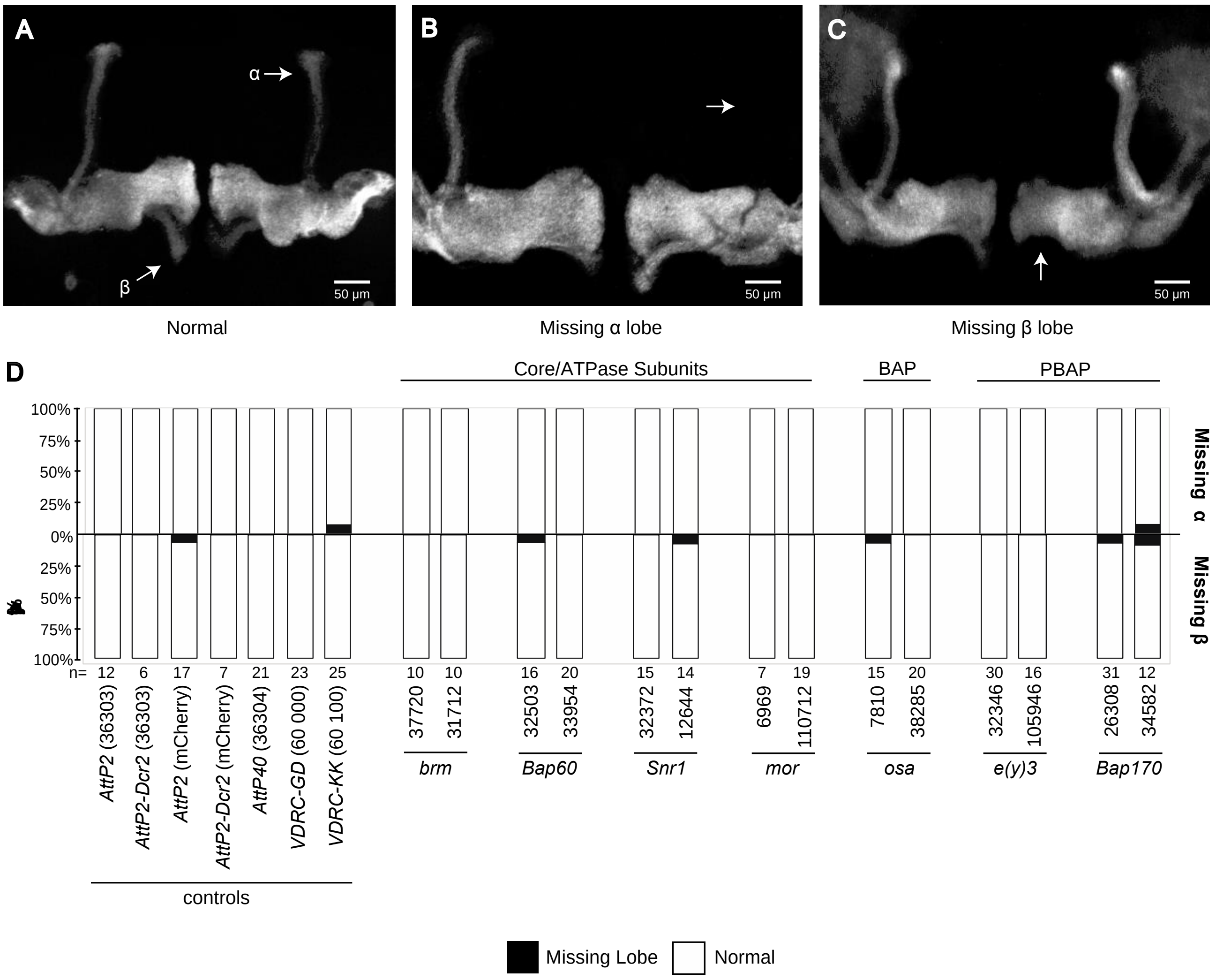

Fig. S2. Missing α and β lobes were observed at low penetrance in SWI/SNF knockdown and control MBs. Confocal projections show (A) normal MB morphology, (B) the missing α-lobe phenotype, and (C) the missing β-lobe phenotype. Arrows indicate the location of normal lobes in panel A and missing lobes in panels B and C. (D) Bar chart showing the total percentage of brains exhibiting the missing α-lobe phenotype (above x-axis) and missing β-lobe phenotype (below x-axis). The total number of flies analyzed for each genotype is indicated.

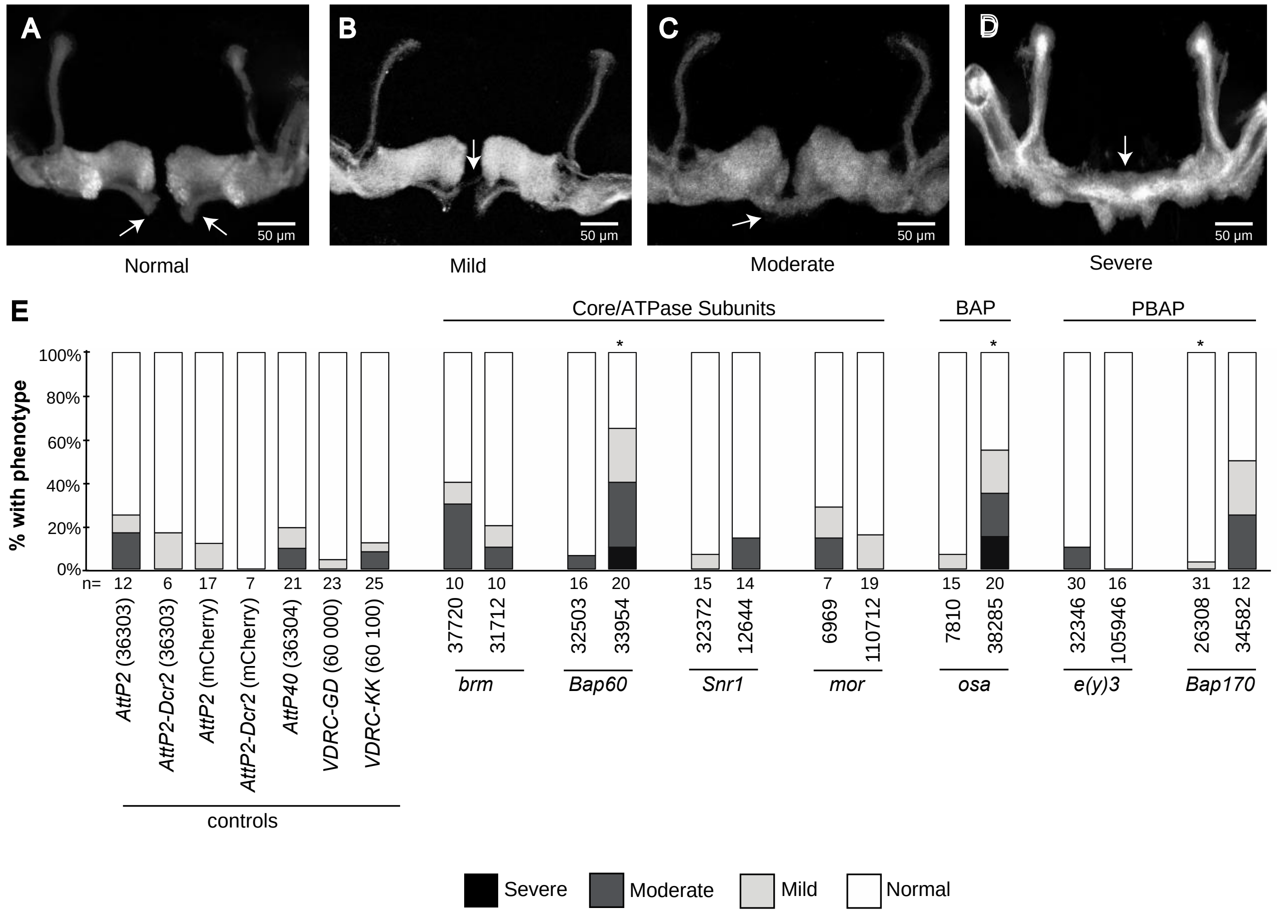

Fig. S3. The appearance of β-lobe fibers crossing the midline was observed at variable penetrance in both knockdown and control MBs. The β-lobe crossing phenotype was qualitatively classified into four categories to account for the observed variation in phenotype severity. Confocal projections show (A) normal MB morphology, as well as the (B) mild, (C) moderate, and (D) severe forms of the β-lobe crossing phenotype. Arrows indicate the normal β-lobe in panel A, and the β-lobe fibers crossing the midline in panels B-D. (E) Bar chart showing the total percentage of brains exhibiting normal MB morphology (white), in addition to the mild (light gray), moderate (dark gray) and severe (black) forms of the β-lobe crossing phenotype. The total number of flies analyzed for each genotype is indicated. *$P < 0.05$; Fisher's exact test, Bonferroni-Dunn test for multiple comparisons.

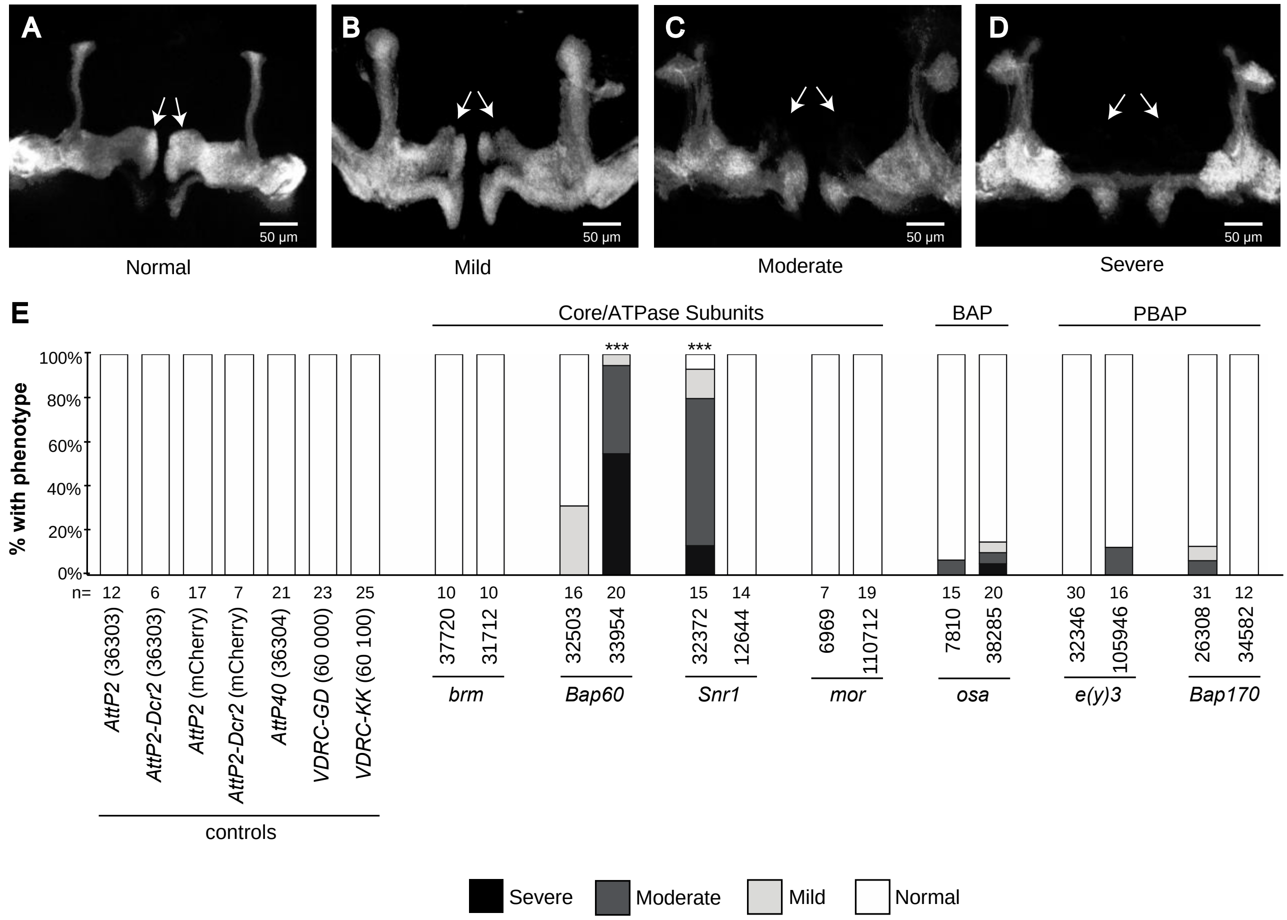

Fig. S4. Quantification of stunted γ-lobe in SWI/SNF knockdown mushroom bodies. (A-D) The appearance of stunted γ-lobe was qualitatively classified into four categories to account for the observed variation in phenotype severity. Confocal projections show representative images for normal MB morphology (A), as well as the mild (B), moderate (C), and severe (D) stunted γ lobe. Arrows indicate the position of the γ-lobe. (B) Bar chart showing the total percentage of brains exhibiting normal (white), mild (light gray), moderate (dark gray) and severe (black) phenotypes. The total number of mushroom bodies analyzed for each genotype is indicated. ****P*<0.001, Fisher's exact test, Bonferroni-Dunn test for multiple comparisons.

Disease Models & Mechanisms • Supplementary information

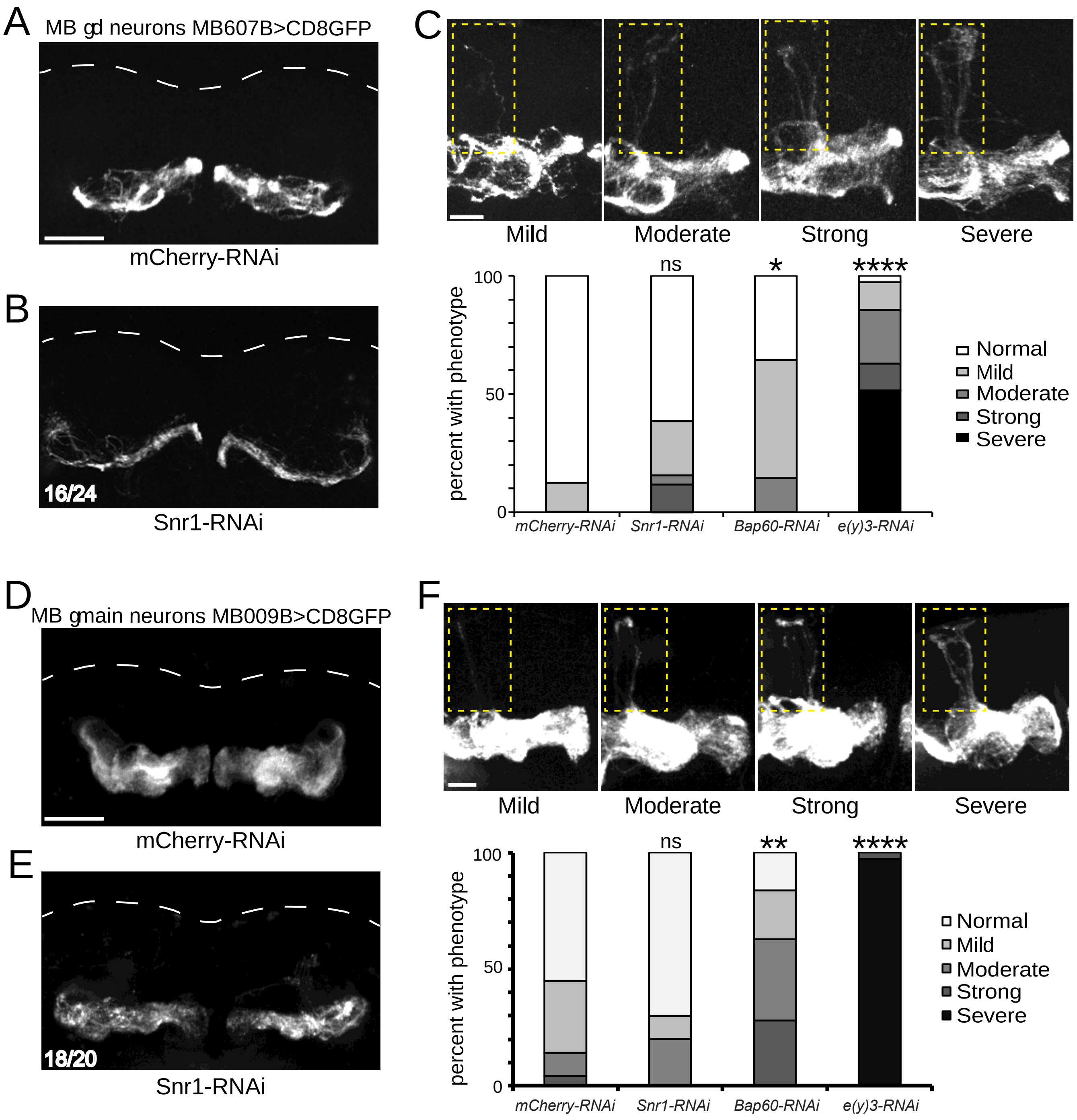

Fig. S5. Identification of extra dorsal projections with established MBγ-specific Split-Gal4 lines. *MB607B-Gal4* (A-C) and *MB009B-Gal4* (D-F) and were used to drive expression of *UAS-mCherry-RNAi* (control), *UAS-Snr1*^{32372}, *UAS-Bap60*^{32503}, *UAS-e(y)3*^{32346}. *MB607B-Gal4* (A) is expressed in the γd neurons while and *MB009B-Gal4* (B) is expressed in the γmain

subset of MB neurons. (C and F) The severity of the extra dorsal projection phenotype was scored by blind observers. For both drivers, knockdown of Bap60 (C n=28, F n=43) and e(y)3 (C n=36, F n=38) caused a significant increase in extra dorsal projection compared to the mCherry-RNAi control (C n=24, F n=49), while Snr1 knockdown (C n=24, F n=20) did not. However, Snr1 knockdown with both drivers (B and E) caused a clear reduction on the volume of MBγ axons present. Numbers in panels B and E indicate the penetrance of the reduced volume phenotype. Scale bars in panels A and D: 50μm. Scale bars in panels C and F: 25μm. White dashed line indicates the dorsal margin of the brain in panels A, B, D, and E. (C,F). Yellow dashed box in panels C and F show the area where extra dorsal projections are observed. $^*P<0.05$, $^{**}P<0.01$,$^{****}P<0.0001$; Kruskal-Wallis test, Dunn's test for multiple comparisons**.**

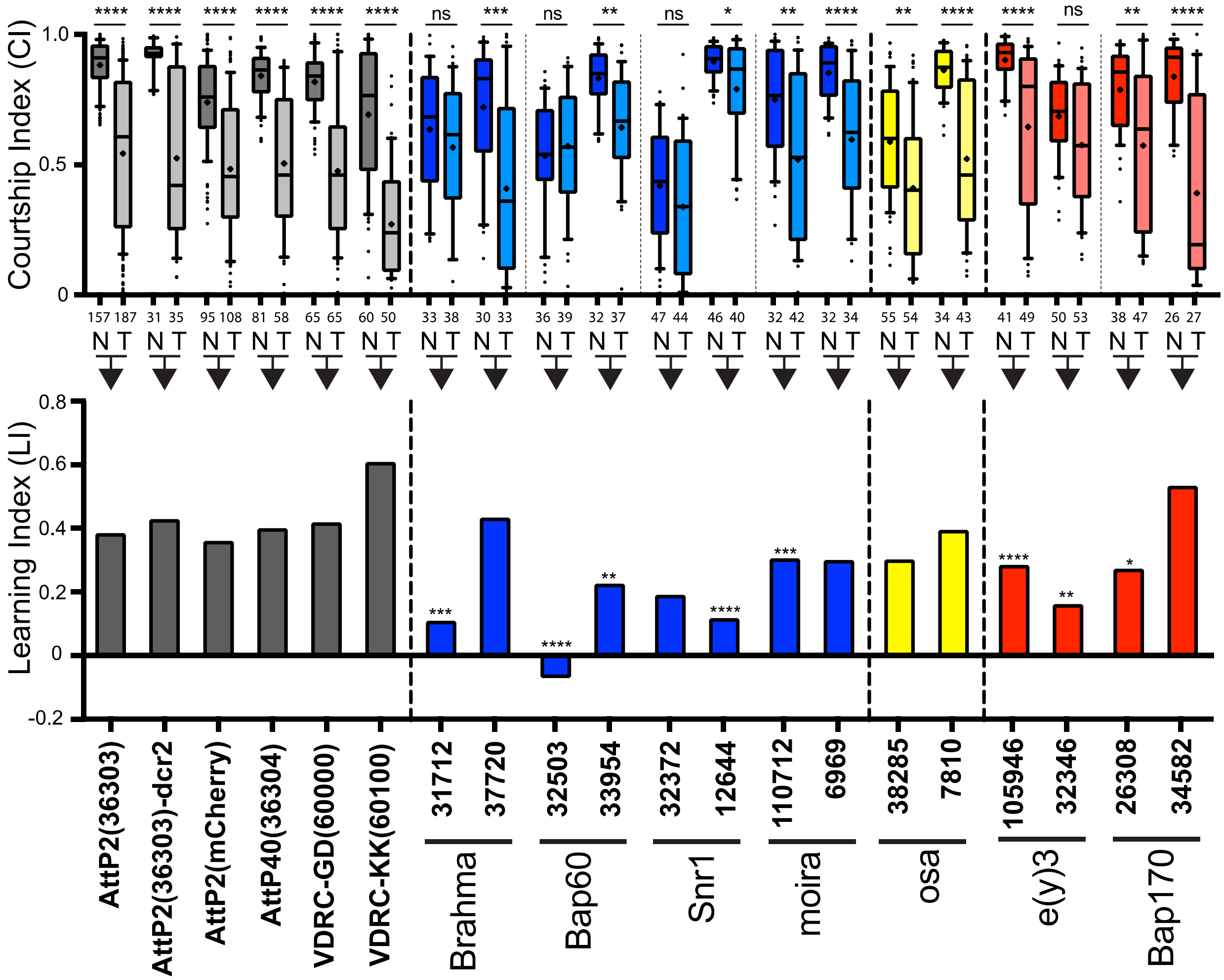

Fig. S6. MB-specific SWI/SNF knockdown causes defects in short-term courtship memory. (A) Box plots indicating the courtship index (CI) for naïve (N) flies and flies that were trained for short-term courtship memory by exposure to sexual rejection for 1 hour (T). Trained and naive flies were tested at the same time, 1 hour after rejection. The number of flies analyzed for each condition is indicated under the box plots. **P*<0.05, ***P*<0.01, *****P*<0.0001; Kruskal Wallis test, Dunn's test for pairwise comparisons. (B) Bar chart showing the learning index (LI) for each genotype [LI = $(CI_{naive}-CI_{trained})/CI_{naive}$]. **P*<0.05, ***P*<0.01, ****P*<0.001, *****P*<0.0001; randomization test, 10,000 bootstrap replicates. Grey bars indicate controls. Purple bars indicate the SWI/SNF core and ATPase subunits. Yellow bars indicate BAP-specific subunits. Red bars indicate PBAP-specific subunits.

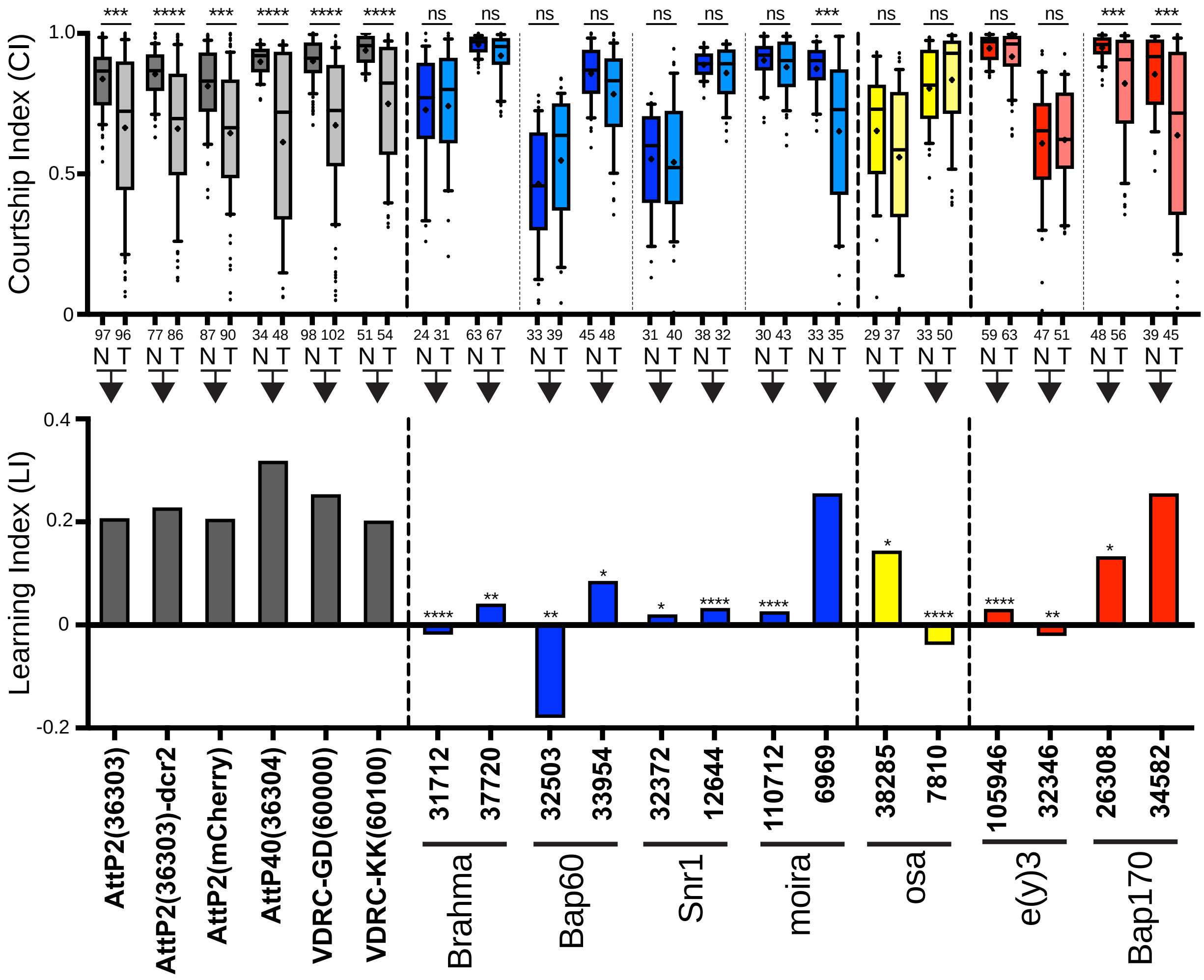

Fig. S7. MB-specific SWI/SNF knockdown causes defects in long-term courtship memory. (A) Box plots indicating the courtship index (CI) for naïve (N) flies and flies that were trained for long-term courtship memory by exposure to sexual rejection for 7 hours (T). Trained and naive flies were tested at the same time, 24 hours after rejection. The number of flies analyzed for each condition is indicated under the box plots. ****P*<0.001, *****P*<0.0001; Kruskal Wallis test, Dunn's test for pairwise comparisons. (B) Bar chart showing the learning index (LI) for each genotype [LI = (CI_{naive}-$CI_{trained}$)/CI_{naive}]. **P*<0.05, ***P*<0.01, *****P*<0.0001; randomization test, 10,000 bootstrap replicates. Grey bars indicate controls. Purple bars indicate the SWI/SNF core and ATPase subunits. Yellow bars indicate BAP-specific subunits. Red bars indicate PBAP-specific subunits.

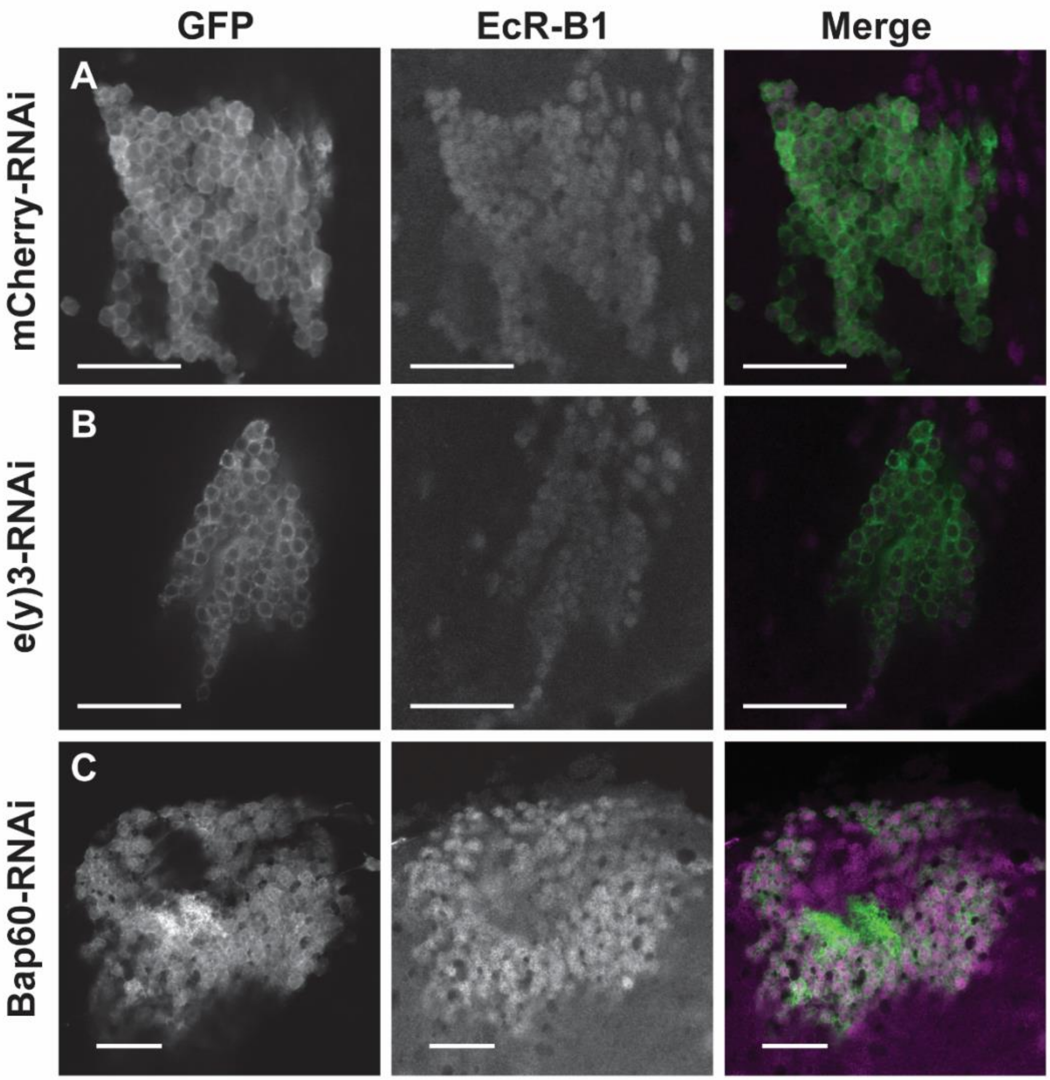

Fig. S8. SWI/SNF knockdown does not affect EcR-B1 expression in third instar larvae MBs. Confocal images showing the MB cell bodies labeled with *R14H06-Gal4* and *UAS-mCD8::GFP* in late third instar larvae (GFP). Controls expressing mCherry RNAi (A) are compared to flies expressing RNAi constructs targeting *e(y)3* [*UAS-e(y)3*^{32346}] (B) and *Bap60* (*UAS-Bap60*^{32503}) (C). EcR-B1 was labeled by immunohistochemistry. Scale bar: 25µm.

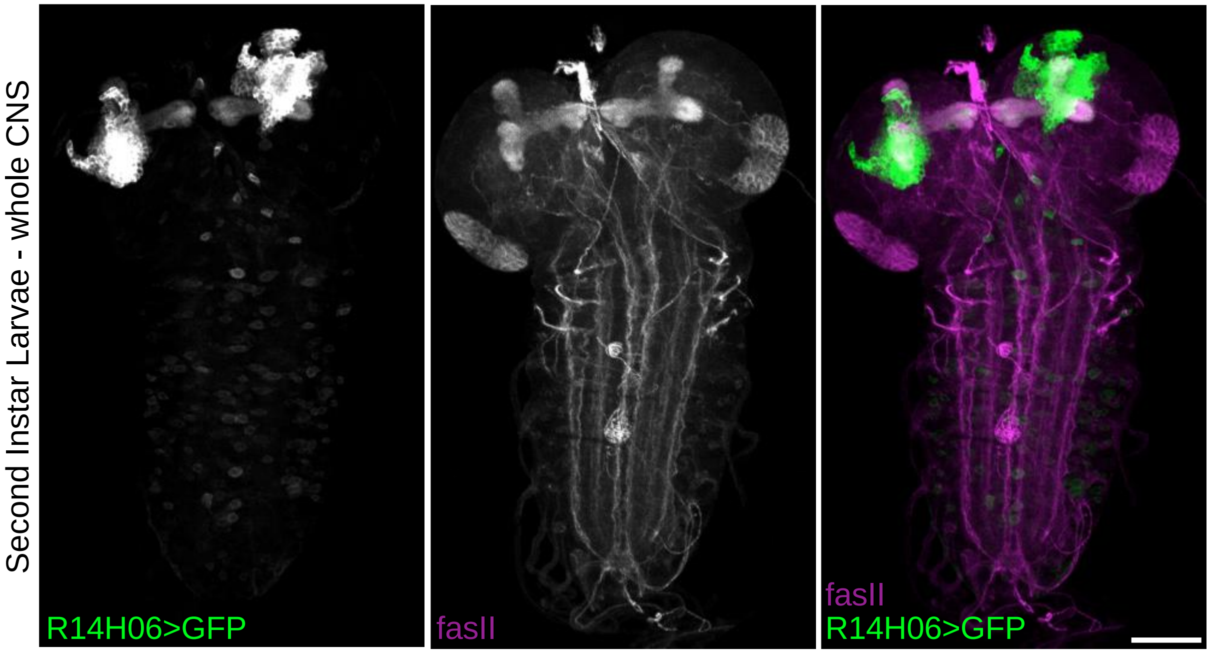

Fig. S9. Expression of *R14H06-Gal4* in the second instar larvae CNS. *UAS-mCD8::GFP* was crossed to *R14H06-Gal4* and the CNS was dissected from second instar larvae. CNSs were fixed and labeled with anti-FasII antibodies and imaged by confocal microscopy. Shown here is a maximum projection of a confocal stack. The MB is clearly and specifically labeled by GFP. Some cells of the ventral nerve chord are also GFP positive. Scale bar: 50μm.

Disease Models & Mechanisms • Supplementary information

Table S1: Evaluation of transgenic RNAi lines using a lethality assay and qPCR.

Gene	RNAi Stock #	Survival Assay		mRNA	
		% Survival[1]	p-value[2]	qPCR[1]	p-value[3]
mCherry	35785	80.2 ± 8.40	< 0.0001		
Bap170	26308	1.70 ± 3.70	< 0.0001	45 ± 3.3	< 0.0001
	34582	0.71 ± 1.15	< 0.0001	44 ± 9.3	< 0.0001
Bap55	24703	5.00 ± 4.40	< 0.0001	NA	NA
	31708	83.3 ± 23.3	0.99	NA	NA
Bap60	32503	0	< 0.0001	48 ± 13	0.0003
	33954	17.8 ± 12.4	< 0.0001	xx	-
brm	31712	0	< 0.0001	46 ± 4.0	< 0.0001
	37720	0.96 ± 0.79	< 0.0001	60 ± 3.0	0.0082
	34520	77.8 ± 15.6	0.99	97 ± 11	0.99
Bap111	35242	3.55 ± 0.97	< 0.0001	284 ± 13	< 0.0001
	26218	1.41 ± 1.96	< 0.0001	85 ± 8.7	0.98
e(y)3	105946	0	< 0.0001	35 ± 3.3	< 0.0001
	32346	0	< 0.0001	15 ± 3.2	< 0.0001
mor	110712	1.20 ± 1.33	< 0.0001	28 ± 2.1	< 0.0001
	6969	0.83 ± 0.93	< 0.0001	22 ± 0.8	< 0.0001
Snr1	32372	4.04 ± 3.92	< 0.0001	xx	-
	12644	53.8 ± 18.8	0.0040	62 ± 3.7	0.017
osa	38285	2.47 ± 1.55	< 0.0001	59 ± 8.0	0.023
	7810	2.13 ± 0.98	< 0.0001	xx	-

[1]± standard error of the mean

[2]ANOVA, Dunnett test for multiple comparisons

[3]ANOVA, Tukey test for multiple comparisons

Disease Models & Mechanisms • Supplementary information

Table S2: RNAi stocks and genetic background controls used in this study

Genetic Background Controls

**Stock name**	**Stock No.**	**Source**	**Genotype**	**Description**
attP2(mCherry)	35785	TRiP	$y^1sc^*v^1$; *P{VALIUM20-mCherry}attP2*	TRiP *attP2* genetic background with an mCherry-RNAi
attP2(36303)	36303	TRiP	y^1v^1;*P{CaryP}attP2*	TRiP *attP2* genetic background control
attP40(36304)	36304	TRiP	y^1v^1;*P{CaryP}attP40*	TRiP *attp40* genetic background control
VDRC-GD(60000)	60000	VDRC	w^{1118}	Isogenic host strain for the VDRC GD RNAi collection
VDRC-KK(60100)	60100	VDRC	*y*, w^{1118}; *P{attP,*y^+, $w^{3`}$*}*	Isogenic host strain for the VDRC KK RNAi collection

RNAi lines

**Gene name**	**Stock No.**	**Source**	**Genotype**	**RNA Hairpin**	**Appropriate Control**
brm	31712	TRiP	y^1v^1;*UAS-brm*^{31712}	Long	*attP2(36303)*
	37720	VDRC	w^{1118};*UAS-brm*^{37720}	Long	*VDRC-GD(60000)*
Bap60	32503	TRiP	$y^1sc^*v^1$;*UAS-Bap60*^{32503}	Short	*attP2* (mCherry)
	33954	TRiP	$y^1sc^*v^1$;*UAS-Bap60*^{33954}	Short	*attP2* (mCherry)
Snr1	32372	TRiP	$y^1sc^*v^1$;*UAS-Snr1*^{32372}	Short	*attP2* (mCherry)
	12644	VDRC	w^{1118};*UAS-Snr1*^{12644}	Long	*VDRC-GD(60000)*
osa	38285	TRiP	$y^1sc^*v^1$;*UAS-osa*^{38285}	Short	*attP40(36304)*
	7810	VDRC	w^{1118};*UAS-osa*^{7810}	Long	*VDRC-GD(60000)*
e(y)3	32346	TRiP	$y^1sc^*v^1$;*UAS-e(y)3*^{32346}	Short	*attP2* (mCherry)
	105946	VDRC	*UAS-e(y)3*^{105946}	Long	*VDRC-KK(60100)*
Bap170	26308	TRiP	y^1v^1;*UAS-Bap170*^{26308}	Long	*attP2(36303)*
	34582	VDRC	w^{1118};*UAS-Bap170*^{34582}*/TM3*	Long	*VDRC-GD(60000)*
mor	6969	VDRC	w^{1118};*UAS-mor*^{6969}	Long	*VDRC-GD(60000)*
	110712	VDRC	*UAS-mor*^{110712}	Long	*VDRC-KK(60100)*

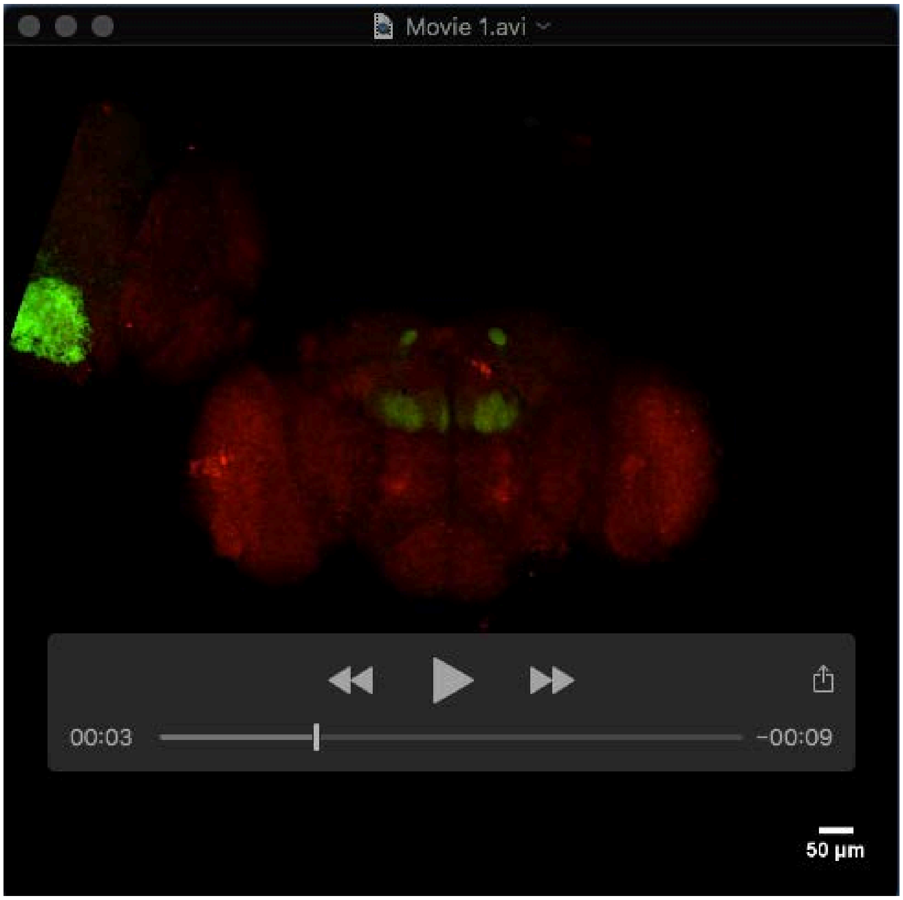

Movie 1. Confocal stack showing the expression domain of *R14H06-Gal4* in the adult *Drosophila* brain. R14H06-Gal4 was used to drive expression of UAS-mCD8::GFP, shown in green. Brains were counterstained in red using the nc82 primary antibody from the Developmental Studies Hybridoma Bank (1:50 dilution) and goat anti-mouse DyLight 594 secondary antibodies (1:400 dilution).
